# Protocol for untargeted lipidomics of human serum using LC-TIMS-PASEF

**DOI:** 10.1016/j.xpro.2026.104607

**Published:** 2026-06-01

**Authors:** Fernanda Sousa Monteiro, Adriana Zardini Buzatto

**Affiliations:** 1 Department of Biological Sciences, University of Calgary, Calgary, AB T2N 1N4, Canada; 2 Alberta Centre for Advanced Diagnostics, University of Calgary, Calgary, AB T2N 1N4, Canada

**Keywords:** Metabolomics, Mass Spectrometry, Protocols in Metabolomics and Lipidomics

## Abstract

Here, we present a protocol for untargeted lipidomics of human serum. We describe steps for project design, sample preparation, and reversed-phase liquid chromatography coupled with trapped ion mobility spectrometry and parallel accumulation serial fragmentation (LC-TIMS-PASEF). We then detail procedures for quality control and data processing. The protocol enables comprehensive lipid profiling from 5 μL of serum, yielding annotations across glycerophospholipids, glycerolipids, sphingolipids, sterols, and fatty acyls. The resulting datasets support statistical analysis and integration with complementary omics approaches.

## Highlights

• Steps for microliter-scale lipid extraction from mammalian serum samples
• Instructions for LC-TIMS-PASEF acquisition of untargeted lipidomics data
• Guidance on quality control using internal standards, pooled samples, and blanks
• Steps for lipid annotation and statistical analysis of lipidomics datasets

## Abstract (editor highlights)

Publisher’s note: Undertaking any experimental protocol requires adherence to local institutional guidelines for laboratory safety and ethics.

## Graphical abstract

**Figure:**
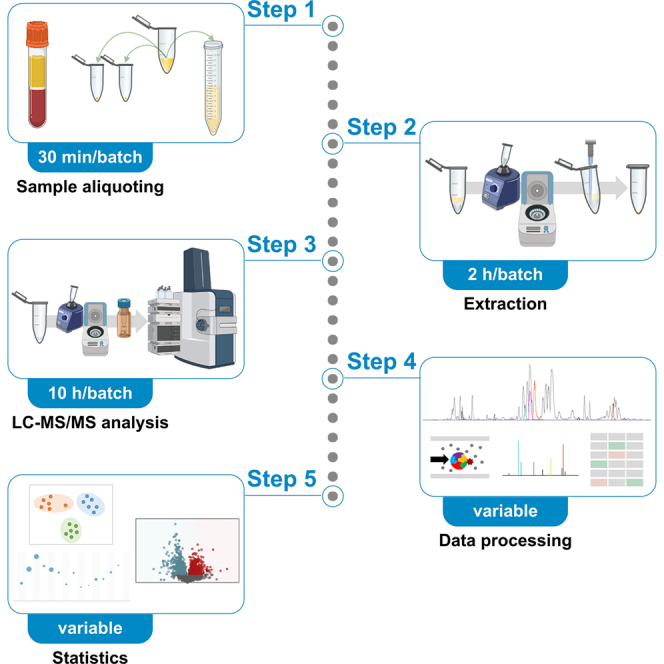

## Before you begin

This protocol describes a workflow for untargeted lipidomics of human serum using reversed-phase liquid chromatography coupled with trapped ion mobility spectrometry and parallel accumulation-serial fragmentation (LC–TIMS–PASEF). It is designed for comprehensive profiling of major lipid categories, including glycerophospholipids, glycerolipids, sphingolipids, sterol lipids, and fatty acyls, and supports relative quantification across biological samples. Mass spectrometry–based lipidomics can detect thousands of features across diverse matrices. The chemical diversity of lipids supports their broad functional roles, but also increases the analytical complexity of studying them. In this workflow, the addition of TIMS provides an orthogonal dimension to support lipid annotation through collision cross-section (CCS) measurements, while PASEF acquisition enables efficient MS/MS fragmentation of co-eluting features. The protocol is suitable for discovery lipidomics, biomarker exploration, and comparative studies across biological states.

In this workflow, serum samples are collected under controlled conditions, randomized, and processed in batches that include pooled quality control (QC) samples and extraction blanks. Lipid extraction from 5 μL of human serum samples is performed using a modified biphasic liquid-liquid extraction protocol derived from the Folch method, with isotopically labeled internal standards added prior to extraction.^1^^,^^2^ Lipid extracts are resuspended in mobile phases for LC-MS/MS analysis using reversed-phase LC–TIMS–PASEF in positive- and negative-ionization modes. The acquired datasets are processed using Bruker MetaboScape 2025b for peak picking, alignment, and initial annotation based on MS/MS spectral matching, supported by collision cross-sections (CCS) and isotope patterns. The data are subsequently refined using a custom pipeline for mass matching, annotation filtering, internal standard normalization, and statistical analysis. The resulting datasets contain annotated lipid features with defined confidence tiers and are suitable for statistical analysis and biological interpretation.

The workflow is specifically developed for LC–TIMS–PASEF acquisition and relies on TIMS separation and PASEF fragmentation. TIMS–PASEF provides orthogonal ion mobility separation compared to conventional LC–MS/MS, improving the characterization of isobaric lipids and supporting annotation via collision cross-section (CCS). PASEF acquisition further increases MS/MS speed and sensitivity by coupling ion accumulation with fragmentation, enhancing fragmentation coverage in complex lipidomes. Core steps of this workflow, such as sample preparation and chromatography, are broadly transferable to other LC–MS platforms with appropriate adaptation, such as mass spectrometry acquisition parameters and data processing steps.

**CRITICAL:** Procurement of human serum must be conducted with approval from an institutional research ethics board by qualified clinical personnel. All procedures involving biological samples, organic solvents, and chemical reagents must be performed with appropriate personal protective equipment (PPE) in accordance with institutional safety and biosafety policies. Human serum should be handled under biosafety level 2 (BSL-2) conditions, or as required by institutional guidelines, prior to organic solvent extraction.

**CRITICAL:** Use glassware dedicated to LC–MS lipidomics for preparation of mobile phases, calibration solutions, and other solvents. Rinse all glassware used in this protocol with three or more portions of LC–MS grade water, followed by LC-MS grade methanol and 2-propanol, to minimize background contamination and leachable interference. Do not use tap water, detergents, or laboratory dishwashers for materials used in this workflow, as residual surfactants and cleaning agents can introduce persistent background signals.

**CRITICAL:** Do not clean laboratory surfaces and floors in areas used for solvent preparation or lipid extraction with commercial cleaning sprays, waxes, or floor-polishing agents, as these products may introduce persistent background contaminants detectable by high-sensitivity LC–MS. Clean benches and surfaces using only 70% ethanol or 2-propanol. Avoid waxing or chemical polishing of floors in lipidomics preparation and analysis areas (or any adjacent areas).

**CRITICAL:** Avoid wearing fragranced cosmetics or hand creams when preparing solvents, mobile phases, or samples for lipidomics. Many personal care products contain lipophilic compounds (e.g., phthalates, siloxanes, fatty esters, ceramides) that are readily detected by LC–MS. Tie back long hair and wear gloves throughout the workflow, replacing them often.

**CRITICAL:** Use the same brand and lot of all reagents and consumables throughout the study to minimize variability. Do not allow mobile phases, calibration solutions, samples, or organic solvents to contact exposed skin or hair, clothes, parafilm, adhesive films, foil, unlined rubber septa or bottle caps, or other laboratory materials that may leach contaminants. Store all prepared solutions exclusively in clean glass bottles with tightly sealed, chemically resistant caps. Bottle caps can leach background compounds (e.g., polysiloxanes), which may appear as recurring features in LC–MS lipidomics datasets. This is particularly relevant for lipidomics workflows, as these compounds are readily extracted and ionized under the conditions used.

### Innovation

This protocol incorporates practical modifications designed to improve reproducibility and robustness in untargeted lipidomics workflows. These include biphasic extraction using dichloromethane and potassium chloride, optimized for microliter-scale serum volumes; integration of ion mobility measurements to support lipid annotation confidence; and a structured quality-control design combining internal standards, pooled quality control (QC) samples, and extraction blanks. The protocol further provides a transparent data-processing routine that combines vendor and custom software, integrates analytical filters and reproducibility criteria, and enables the generation of lipidomics datasets for downstream analysis from complex biological matrices.

## Key resources table

**Table:** 

REAGENT or RESOURCE	SOURCE	IDENTIFIER
**Biological samples**		

Human Serum	Millipore Sigma (or study subjects)	Cat. No. S1-100ML

**Chemicals, peptides, and recombinant proteins**		

Dichloromethane, HPLC grade	Fisher Scientific	Cat. No. AC364230025
Methanol, LC–MS grade	Fisher Scientific	Cat. No. A456-4
Water, LC–MS grade	Fisher Scientific	Cat. No. W6-4
Isopropanol, LC–MS grade	Sigma-Aldrich	Cat. No. 1027814000
Acetonitrile, LC–MS grade	Fisher Scientific	Cat. No. A955-4
Ammonium formate, LC/MS Grade	Fisher Scientific	Cat. No. A115-50
Formic acid, LC/MS Grade	Fisher Scientific	Cat. No. A117-50
SPLASH™ LIPIDOMIX™ Mass Spec Standard	Avanti Research (Millipore-Sigma)	Cat. No. 330707-1EA
Potassium Chloride (KCl), USP/FCC Grade	Fisher Scientific	Cat. No. P330-500
ESI-L LCMS Tuning Solution (Including ESI-TOF)	Agilent Technologies	Cat. No. G1969-85000

**Deposited data**		

Example dataset	Metabolights: MTBLS14002	https://www.ebi.ac.uk/metabolights/MTBLS14002

**Software and algorithms**		

MetaboScape 2025b	Bruker Daltonics	Cat. No. 1878412
DataAnalysis 6.1	Bruker Daltonics	N/A
Compass HyStar 6.3	Bruker Daltonics	N/A
Lipid databases: MoNA LipidBlast 2022^3^ MoNA HMDB^4^^,^^5^	Mass Bank of North America	https://mona.fiehnlab.ucdavis.edu/
Lipid database: LIPID MAPS	LIPID MAPS	https://www.lipidmaps.org/
Lipidomics data processing routine; suggested: Buzatto Research Group (BRG) LipidQuest	Buzatto Research Group, University of Calgary, Canada	GitHub: https://github.com/Buzattoresearch/BRG-LipidQuest https://doi.org/10.5281/zenodo.19545764
Python 3.9	Python Software Foundation	https://www.python.org/
Python package: Numpy 1.26.4	N/A	https://numpy.org/
Python package: Pandas 2.3.0	N/A	https://pandas.pydata.org/
Python package: Matplotlib 3.9.4	N/A	https://matplotlib.org/
Python package: Seaborn 0.13.2	N/A	https://seaborn.pydata.org/
Python package: Scipy 1.13.1	N/A	https://scipy.org/
Python package: Statsmodels 0.14.4	N/A	https://www.statsmodels.org/stable/index.html

**Other**		

1290 Infinity III LC System, including InfinityLab Assist Hub, 1290 High Speed Pump, 1290 Multisampler, and 1290 MCT	Agilent Technologies	Cat. No. G7180A, G7120A, G7167B, G7116B
timsTOF Pro2 Mass Spectrometer with VIP HESI ion source for LC coupling	Bruker Daltonics	Cat. No. L-1910000, 1884274
ACQUITY CSH C18 column 130Å, 2.1 mm × 100 mm, 1.7 μm	Waters Corporation	Cat. No. 186005293
Refrigerated centrifuge with rotor for 1.5 mL microcentrifuge vials capable of reaching 14,000 × *g* or above (e.g., Eppendorf 5430 R with rotor FA-45-30-11)	Eppendorf	Cat. No. 022620623
Refrigerator (4°C)	N/A	N/A
Freezer (−80°C)	N/A	N/A
Vortex mixer	Fisher Scientific	Cat. No. 02-215-414
Mini-centrifuge	Fisher Scientific	Cat. No. 12006901
Pipette 100–1000 μL	Gilson	Cat. No. FA10006M
Pipette 20–200 μL	Gilson	Cat. No. FA10005M
Pipette 2–20 μL	Gilson	Cat. No. FA10003M
Pipette tips, Sterile, Filtered (100–1000 μL)	VWR	Cat. No. 76322-154
Pipette tips, Sterile, Filtered (20–200 μL)	VWR	Cat. No. 76322-150
Pipette tips, Sterile, Filtered (1–20 μL)	VWR	Cat. No. 76322-134
Vacuum concentrator (Vacufuge Plus Centrifuge Concentrator)	Eppendorf	Cat. No. 05414174
1.5 mL Safe-Lock Microcentrifuge tubes	Eppendorf	Cat. No. 022363204
2 mL amber, screw-top vials	Agilent Technologies	Cat. No. 5182-0716
Screw caps with bonded blue PTFE/red silicone septa	Agilent Technologies	Cat. No. 5190-7024
300 μL polypropylene vial inserts with polymer feet	Agilent Technologies	Cat. No. 5182-0549
2 mL amber, screw-top vials with 300 μL fixed glass inserts	Agilent Technologies	Cat. No. 5188-6592
20 mL clear glass vials with PTFE-lined closed-top caps	Thermo Scientific	Cat. No. C1260020
1 L storage glass bottles	Corning	Cat. No. 13991 L
Vacuum filtration assembly with fritted glass support	DWK Life Sciences	Cat. No. 419380
PTFE membrane filter, 0.2 μm pore size	Millipore Sigma	Cat. No. JGWP04700

## Materials and equipment

**Table:** 0.2 M Potassium Chloride Solution

Reagent	Final concentration	Amount
Potassium Chloride (analytical grade or above)	0.2 M	1.491 g
Water (LC-MS grade)	N/A	Bring to 100 mL

***Note:*** The 0.2M potassium chloride solution is used for liquid-liquid extractions of lipids from serum samples. Potassium chloride is a low-hazard chemical; handle using standard PPE, including a laboratory coat, non-powdered nitrile gloves, and safety glasses. Weigh potassium chloride, then add 100 mL LC–MS grade water, and dissolve completely. Filtration is not required if high-purity reagents are used. The 0.2M potassium chloride solution can be stored in a sealed glass bottle for up to 4 weeks at 4°C.

**Table:** 3.33 M Ammonium Formate Stock Solution

Reagent	Final concentration	Amount
Ammonium formate (analytical grade or above)	3.33 M	10.4988 g
Water (LC-MS grade)	N/A	Bring to 50 mL

***Note:*** The 3.33M ammonium formate stock solution is used to prepare mobile phases. Ammonium formate is an irritant. Avoid inhalation and handle with a laboratory coat, non-powdered nitrile gloves, and safety glasses. It is hygroscopic and readily absorbs atmospheric moisture; minimize bottle open time and store tightly sealed, ideally in a desiccated environment. Dissolve ammonium formate completely in LC-MS-grade water, then adjust the final volume to 50 mL with LC-MS grade water. Store the stock solution at 4°C in a tightly sealed glass container for up to 30 days. Discard the solution after 30 days, as ammonium formate gradually decomposes, leading to concentration drift and altered chromatographic performance. Under the conditions described, routine pH monitoring is not required for this workflow.

**Table:** Mobile Phase A (MPA): 10 mM ammonium formate in 40:35:25 methanol/acetonitrile/water (v/v/v)

Reagent	Final concentration	Amount
Water (LC-MS grade)	24.7%	247 mL
3.33 M Ammonium Formate Stock Solution	10 mM	3 mL
Methanol (LC-MS grade)	40.0% (v/v)	400 mL
Acetonitrile (LC-MS grade)	35.0% (v/v)	350 mL

***Note:*** Mobile phase A is used for resuspension and chromatographic separation of lipid extracts. Measure all solvents volumetrically using graduated cylinders and, when applicable, calibrated pipettes. Add 3 mL of the 3.33M ammonium formate stock solution to 247 mL of LC-MS grade water in a clean 1 L glass bottle with a PTFE-lined cap, or in a volumetric flask, and mix thoroughly. Add 400 mL of LC-MS grade methanol using a graduated cylinder. Add LC-MS grade acetonitrile to a final volume of 1 L. Filter through a 0.2 μm PTFE membrane using a vacuum assembly with fritted glass support. Store between 20°C and 24°C for up to one week in a tightly sealed glass bottle with a solvent-resistant cap.

**Table:** Mobile Phase B (MPB): 10 mM ammonium formate in 96:3:1 2-propanol/acetonitrile/water (v/v/v)

Reagent	Final concentration	Amount
Water (LC-MS grade)	0.7%	7 mL
3.33 M Ammonium Formate Stock Solution	10 mM	3 mL
Acetonitrile (LC-MS grade)	3.0% (v/v)	30 mL
2-propanol (LC-MS grade)	96.0% (v/v)	960 mL

***Note:*** Mobile phase B is used for resuspension and chromatographic separation of lipid extracts. Add 3.0 mL of the 3.33M ammonium formate stock solution to 7.0 mL of LC-MS grade water in a clean 1 L glass bottle with a PTFE-lined cap, or in a 1 L volumetric flask, and mix thoroughly. Add 30 mL of LC-MS grade acetonitrile using a graduated cylinder. Add LC-MS-grade 2-propanol to a final volume of 1 L, then shake vigorously and release pressure frequently. Filter through a 0.2 μm PTFE membrane using a vacuum assembly with fritted glass support. Store between 20°C and 24°C for up to one week in a tightly sealed glass bottle with a solvent-resistant cap.

**CRITICAL:** Replace mobile phase bottles entirely every 7 days rather than topping them off to prevent contamination, solvent-composition drift, and ammonium formate degradation. Discard mobile phases if turbidity, precipitate, or retention-time instability is observed. Prepare mobile phases with precise solvent ratios and ammonium formate concentration, as small deviations can alter retention times and ionization efficiency.

**CRITICAL:** Do not attempt to dissolve ammonium formate directly in methanol, acetonitrile, or 2-propanol, as incomplete dissolution or delayed precipitation may occur. Dissolve completely in water before adding organic solvents.

**CRITICAL:** 2-Propanol, methanol, and acetonitrile are flammable organic solvents and should be handled away from ignition sources. Methanol is toxic by ingestion, inhalation, and skin absorption. Use appropriate PPE (laboratory coat, non-powdered nitrile gloves, and safety glasses) and handle solvents in a well-ventilated area or fume hood.

**Table:** Needle wash: (v/v/v)

Reagent	Final concentration	Amount
Water (LC-MS grade)	22% (v/v)	220 mL
Methanol (LC-MS grade)	36% (v/v)	360 mL
Acetonitrile (LC-MS grade)	32% (v/v)	320 mL
2-propanol (LC-MS grade)	10% (v/v)	100 mL

***Note:*** The needle wash solution is used to clean the external surface of the LC injector needle between injections to minimize carryover, reduce background contamination, and improve injection reproducibility. Mix solvents in a clean 1 L glass bottle with a PTFE-lined cap, or in a volumetric flask, and shake vigorously. Store between 20°C and 24°C in a glass bottle for up to 30 days.

**Table:** 10 mM Sodium Formate Stock Solution

Reagent	Final concentration	Amount
Sodium Hydroxide (analytical grade or above)	10 mM	0.040 g
Formic acid (chromatography grade or above)	0.2% (v/v)	200.0 μL
Water (LC-MS grade)	50% (v/v)	50.0 mL
2-propanol (LC-MS grade)	≈50%	Bring to 100 mL

***Note:*** A 10 mM sodium formate solution in 1:1 water/2-propanol (v/v) is used for mass calibration. Dissolve 0.040 g sodium hydroxide in 50 mL LC–MS grade water. Do not dissolve sodium hydroxide directly in a volumetric flask, as the dissolution is exothermic and can damage the glass. Once the sodium hydroxide is fully dissolved, add 200 μL formic acid slowly while mixing. Bring the solution to a final volume of 100 mL with LC–MS grade 2-propanol and mix thoroughly. Store the solution in a tightly sealed container with a PTFE-lined cap at 4°C for up to 4 weeks. Shake well before use.

**CRITICAL:** Formic acid is corrosive and volatile; handle in a certified chemical fume hood or well-ventilated area. Sodium hydroxide is corrosive and causes severe skin and eye burns. Wear laboratory coat, non-powdered nitrile gloves, and safety glasses when handling either reagent. Do not store concentrated sodium hydroxide solutions in glass bottles, as strong alkali can etch glass surfaces. The diluted 10 mM sodium formate solution described here is suitable for storage in glass containers at 4°C.

### LC conditions for lipidomics analysis

***Note:*** This workflow was designed using an Agilent 1290 Infinity III UHPLC system (Agilent Technologies, Santa Clara, CA, United States). Use an ACQUITY UPLC CSH C18 column (130Å, 2.1 × 100 mm, 1.7 μm; Waters Corporation, Milford, MA, USA), with the column compartment temperature maintained at 50°C. Prepare a gradient elution method following the program described in Table 1. Set the injector draw speed to 60 μL/min, the eject speed to 300 μL/min, the wait time after draw to 0.5 s, the needle height offset to 0.5 mm (no vial bottom sensing), and use standard wash (i.e., flush the port for 5 s) during injections. The total run time for this method is 20 min per injection, including column re-equilibration.

***Note:*** Run a longer needle wash cycle (30 s needle wash, followed by 60 s 100% 2-propanol, and another 30 s needle wash) once daily. Rinse the LC-MS system weekly with 1:1 methanol/water and 100% 2-propanol at 0.100 mL/min for approximately 1 h each to reduce lipid accumulation and prevent column, sprayer, and/or tubing contamination and blockage.

**CRITICAL:** Assess chromatographic performance weekly or whenever mobile phases are replaced. Record system backpressure at 10% mobile phase B (0.350 mL/min), 50% mobile phase B (0.350 mL/min), and 99% mobile phase B (0.280 mL/min) at 50°C. Document baseline signal intensity under low- and high-organic conditions. Abrupt or progressive changes in backpressure (≥±10%) may indicate column fouling, system blockage, or leaks. Baseline instability may indicate solvent contamination or mobile-phase degradation. Prepare fresh mobile phases if solvent instability is suspected (see troubleshooting – problem 2 and problem 4).

**CRITICAL:** Do not operate the LC system at 99% mobile phase B with flow rates above 0.280 mL/min or column temperatures below 50°C. High viscosity under these conditions can generate excessive backpressure (>1,000 bar) and damage the column or LC system.

### Mass spectrometry parameters

***Note:*** This workflow was designed with a Bruker timsTOF Pro2 mass spectrometer (Bruker Daltonics, Billerica, MA, United States) equipped with a 6-port divert valve and a Vacuum Insulated Probe-Heated ElectroSpray Ionization (VIP-HESI) source. Mass spectrometry acquisition parameters are described in Table 2.

***Note:*** Clean the ionization source weekly with 1:1 water/2-propanol (v/v) to minimize background contamination. Perform mass and mobility calibration every 48 h. Clean the mass spectrometer’s glass capillary every 3 months with 1:1 water/methanol and 2-propanol, following the manufacturer’s instructions. Equilibrate the system and the analytical column to the initial mobile-phase conditions, temperatures, and voltages for at least 15 min before injecting samples. Check pressures and baseline intensity whenever mobile phases are replaced.

**CRITICAL:** Calibrate the Bruker timsTOF Pro2 mass spectrometer for mass accuracy and ion mobility every other day during active use using a 2:1 (v/v) mixture of Agilent ESI-L LC-MS Tuning Solution and 10 mM sodium formate solution. For routine instrument calibration, deliver the calibration mixture via syringe pump at 50 μL/min directly to the ion source. Mass and mobility calibration performance is indicated by a quality score in Bruker’s timsControl software. Do not acquire data if the score is below 100% or if the standard deviation is higher than 0.50 ppm for mass or mobility accuracy. Monitor instrument performance by recording calibrant signal intensity and tracking the standard deviation of mass and ion mobility collision cross-section (CCS) values across calibration events. Investigate significant drift in mass or mobility accuracy before proceeding with sample analysis. During injections, set the flow of calibrant mixture to 40 μL/h using a 6-port valve with a 20 μL loop set to waste. Run the LC mobile phase through the loop during the initial 0.3 min of each injection, pushing the calibrant mixture into the ion source, to create a calibration segment.

***Note:*** Sodium formate forms a series of cluster ions with well-defined masses, enabling accurate mass calibration across a wide m/z range (<2000 m/z). However, sodium salts are non-volatile; prolonged infusion or high flow rates can contaminate the ion source. Use low infusion rates and divert the calibration solution to waste whenever possible.

***Note:*** This workflow was developed using a Bruker timsTOF Pro2 mass spectrometer coupled to an Agilent 1290 Infinity III UHPLC system. Other LC–MS platforms may be used, but require adjustments to chromatographic gradients, ionization settings, and MS acquisition parameters.

## Step-by-step method details

This section describes the step-by-step workflow for untargeted lipidomics of human serum samples, from experimental planning and batch organization through lipid extraction, LC–MS acquisition, and data processing.

### Serum collection

**Timing: Hours to days, depending on study size**

This section describes the general procedure for collecting human serum.

***Note:*** A minimum of five biological replicates per experimental group is recommended, but sample size must be determined *a priori* through appropriate biostatistical planning, such as power analysis, to ensure adequate statistical power. Technical replicates do not replace biological replicates. Blood draws should be performed exclusively by specialized clinical personnel with approval of institutional ethics committees.

**CRITICAL:** Carefully consider individual diversity in studies involving human or animal subjects, as physiological state and environmental exposures strongly influence the lipidome. Match variables that may introduce systematic bias between groups, including age, sex, body mass index (BMI), and medication use. Lipids respond dynamically to physiological variables such as diet, exercise, comorbidities, and microbiome.

1. Collect venous blood by clean venipuncture using tubes containing a clot activator (e.g., thrombin or silicate).

**CRITICAL:** Minimize tourniquet time and avoid mechanical stress during collection to reduce hemolysis, as erythrocyte lysis releases membrane lipids and can substantially distort lipid profiles. Use the same tube type, manufacturer, and lot number throughout the study.

2. Allow blood to clot upright at 20°C–24°C for 30 min.
3. Centrifuge at 1,500 × *g* (relative centrifugal force, RCF) for 10 min at 4°C.
4. Carefully transfer the clear serum layer to low-bind, colorless 1.5 mL polypropylene tubes.
5. Visually inspect samples and exclude those with evident hemolysis (red or brown tones).
6. Snap-freeze immediately and store at −80°C.

***Note:*** Once frozen, samples must not undergo additional freeze–thaw cycles, as repeated freezing and thawing alter lipid composition.

7. Record preanalytical variables, including fasting status, time of collection, clotting agent and duration, centrifugation conditions, brand and lot of tubes, and freezing time.

**CRITICAL:** To avoid potential infection risk with bloodborne pathogens, perform all work with appropriate personal protection equipment and follow institutional biosafety requirements.

***Note:*** Human serum can generally be stored at −80°C for at least one year if samples are frozen quickly after collection, protected from light and oxygen, and not thawed. Minimal lipidome drift is expected for polyunsaturated fatty acids, oxidized lipids, and lysophospholipids. Storage at −20°C is not recommended due to increased enzymatic activity and oxidation. Minimize exposure to light and heat during processing. Avoid repeated freeze–thaw cycles.

**CRITICAL:** Collect and store all samples within a project using identical procedures to minimize *ex vivo* variability.

**Pause point:** Collected serum samples can be stored at −80°C for up to one year.

### Project design and experimental planning

**Timing: 1–2 h**

This section describes the pre-analytical steps required for appropriate experimental planning.

***Note:*** Quality control is implemented at three levels: (1) addition of deuterated lipid internal standards to every sample prior to extraction, (2) inclusion of a pooled QC mixture composed of equal aliquots from all study samples within the project, and (3) inclusion of extraction blanks prepared using water in place of serum to monitor background contamination. Use the same pooled QC mixture throughout the entire project to ensure longitudinal comparability. To minimize variability arising from extended extraction windows, limit processing to at most 24 extractions (two batches of 10 samples, one QC, and one extraction blank) per day.

**CRITICAL:** Include in the experimental plan three independent QC extractions and three independent blank extractions on the first day to establish baseline analytical stability. Each subsequent batch contains 10 samples, one QC extraction, and one blank.

***Note:*** A maximum of two batches can be processed and injected per day (approximately 20–22 h of total acquisition time). The remaining time within a day is used for system suitability checks, calibration, and cleaning procedures. Each batch consists of 10 study samples, one pooled QC extract, and one extraction blank. All extracts, including QC and blank samples, are analyzed in both positive- and negative-ionization modes, as different lipid classes ionize preferentially under different conditions. Each extract is therefore injected once in positive and once in negative ionization, resulting in 24 injections per batch (10 sample extracts, one QC, and one blank, each analyzed in two ionization modes).

**CRITICAL:** Lipid extraction and LC–MS injection order must be randomized independently of biological grouping to minimize batch effects and systematic bias across large cohorts. Each batch must include a pooled quality control sample composed of equal aliquots from all study samples and an extraction blank containing all reagents except biological material.

8. Prepare a complete sample list including all study samples, their experimental group assignments, and metadata.
9. Randomize all samples independently of biological grouping.

**CRITICAL:** Do not extract samples grouped by condition or phenotype. Maintain randomization across extraction and injection order to minimize batch effects and systematic bias. Randomization can be performed using any appropriate method. For example, assign each sample a numerical order (1 to n), generate a randomized sequence using spreadsheet software (e.g., RANDBETWEEN function in Excel), and use this order for extraction and LC–MS analysis.

10. Divide the randomized samples into batches. Each batch must include:
  a. One pooled QC composed of equal-sized aliquots from all study samples in the project.
  b. One extraction blank, prepared using water in place of serum.
  c. A maximum of 10 randomized study samples.

***Note:*** Extraction blanks monitor background contamination arising from solvents, consumables, and sample handling. Evaluate blank-derived features during downstream checks and data filtering.

11. Calculate the total volume of pooled QC required for the entire project, including three initial QC extractions and one QC aliquot per sample batch.

totalvolumeofpooledQC=(totalnumberofbatches+3)×5μL

12. Determine the required volume from each sample for the pooled QC by dividing the total QC volume by the number of samples.

minimumvolume=1.25×(totalnumberofbatches+3)×5μL

***Note:*** Prepare the pooled QC once during sample aliquoting and use it consistently throughout the study. If sample volume allows, prepare at least a twofold excess of pooled QC to enable reinjection, reanalysis, or additional quality-control injections as needed.

13. Calculate the total volume of internal standard mixture required for the project. Each sample, QC, and blank must receive the same volume of internal standard mixture (4 μL).

totalvolumeofinternalstandardmixture=[totalnumberofsamples+2×(totalnumberofbatches+3)]×4μL

***Note:*** Use a single manufacturer and lot number for the internal standard mixture across all samples in a project to ensure quantitative consistency. Ensure a twofold excess of internal standard mixture from the same manufacturer and lot number is available before starting extractions.

***Note:*** This workflow uses the Avanti SPLASH Lipidomix Mass Spec Standard (Avanti Research) as the internal standard mixture. Alternative options, such as in-house mixtures of isotopically labelled internal standards, may be used but require optimization of concentrations.

14. Calculate the total amount of consumables (pipette tips, microcentrifuge tubes, LC–MS vials, inserts, vial caps) and solvents required to complete all batches.
15. Confirm the availability of all required materials, solvents, and reagents from the same manufacturer and lot number to reduce variability.

***Note:*** This protocol has been optimized for lipid extraction from 5 μL of serum. The workflow has also been successfully applied to plasma and other biological fluids. Solvent volumes may be scaled proportionally to accommodate different sample volumes. When scaling, maintain a total organic solvent volume equivalent to 100 μL per μL of sample, consisting of methanol and dichloromethane in a 1:2 (v/v) ratio. The Avanti SPLASH Lipidomix internal standard mixture is supplied in methanol. The volume of internal standard solution must therefore be considered as part of the methanol fraction when maintaining the solvent composition. For example, use 500 μL of 1:2 methanol/dichloromethane (4 μL of internal standard mixture, 163 μL methanol, 333 μL dichloromethane) for 5 μL of serum; or reduce to 400 μL (3.2 μL of internal standard mixture, 130.0 μL methanol, 267 μL dichloromethane) for 4 μL of serum. Maintain a final resuspension dilution factor of 10× (i.e., 5 μL of mobile phase B, MPB, and 5 μL of mobile phase A, MPA, per μL of sample).

### Aliquoting and storage of serum samples

**Timing: Hours to days, depending on study size**

**Figure 1:**
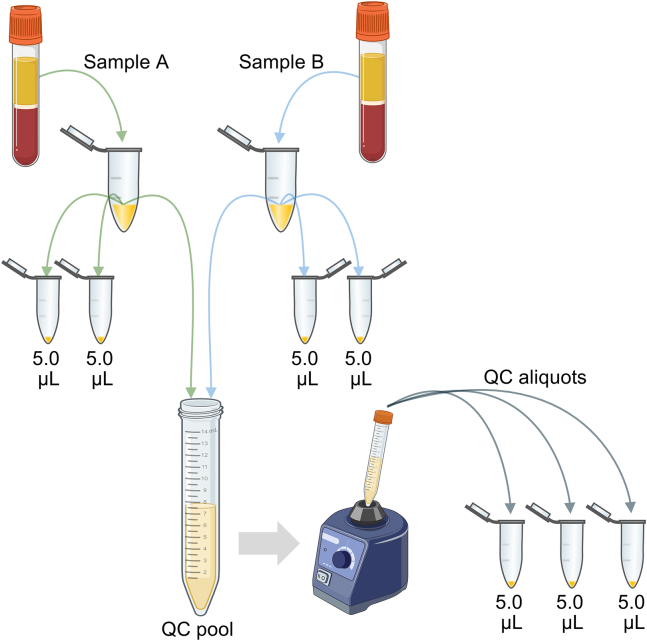
Sample aliquoting protocol After clotting, blood serum is collected into microcentrifuge tubes and split into single-use aliquots. A quality control (QC) pool is simultaneously prepared from a small amount of each sample. The total QC pool volume may exceed the capacity of standard microcentrifuge tubes. Pool samples in a high-quality conical tube (e.g., Falcon) or clean glass bottle (solvent-rinsed, low-contamination), then mix and split into microcentrifuge tubes prior to extraction. Do not perform extractions in conical tubes. One QC aliquot and one blank extract are prepared with each batch of 10 samples. Icons were obtained from BioRender.

This step describes the aliquoting procedure for serum samples (Figure 1).

**CRITICAL:** Many lipids are chemically labile and degrade rapidly under ambient conditions. Oxidation, hydrolysis, and enzymatic activity can alter lipid composition, leading to artifactual increases in lysophospholipids, diacylglycerols, and oxidized lipid species, along with reduced levels of polyunsaturated fatty acids. Minimize exposure to heat, oxygen, and light. Aliquot serum into multiple single-use volumes and prepare and aliquot the pooled QC mixture at the same time to prevent additional freeze-thaw cycles. To ensure consistent handling, all study samples and pooled QC must be aliquoted prior to extraction.

***Note:*** Antioxidants such as butylated hydroxytoluene (BHT) have been used to limit *ex vivo* oxidation; however, they are not universally compatible with mass spectrometry, may introduce background signals, and exhibit matrix- and concentration-dependent effects.^6^^,^^7^^,^^8^^,^^9^^,^^10^ Unless specific oxidation-prone lipid subclasses are under investigation, rapid processing is preferable to routine antioxidant supplementation.

16. Clearly label sufficient 1.5 mL microcentrifuge tubes for all study samples and QC aliquots.
17. Label a clean, low-contamination container (e.g., 15 mL conical tube or solvent-rinsed glass bottle) for the QC pool, ensuring sufficient capacity for the total pooled volume.

***Note:*** This container is used only during sample aliquoting. The pooled QC is subsequently transferred to microcentrifuge tubes for extraction; lipid extractions are not performed in conical tubes.

18. Thaw the first randomized batch of 10 serum samples at 4°C. Maintain the randomized order to minimize batch effects.

**CRITICAL:** Biological samples contain active enzymes and labile lipids. Thaw samples at 4°C and limit thaw time to the minimum required for aliquoting. Aliquot each sample into at least two portions: one for extraction and one reserved for potential reanalysis. All samples must undergo the same number of freeze–thaw cycles, under identical conditions and timing, to prevent systematic bias (see troubleshooting – problem 1).

19. Once thawed, vortex each sample for 30 seconds to ensure complete homogenization.
20. Aliquot 5 μL of serum into pre-labelled microcentrifuge tubes designated for extraction, using a new, pre-wetted pipette tip for each transfer.

***Note:*** Use calibrated micropipettes and low-retention tips for accurate handling of microliter-scale volumes.

**CRITICAL:** Use a new pipette tip for every transfer, even when transferring the same sample or solvent. Reuse of pipette tips can cause cross-contamination and inaccurate volumes due to solvent evaporation or adsorption. Pre-wet each pipette tip with the solution or sample to be transferred before dispensing to improve volumetric accuracy and precision. Pre-wetting consists of aspirating and dispensing the solution with the pipette tip two to three times prior to the final measured transfer. Regularly calibrate pipettes for accuracy and precision in accordance with institutional quality control procedures and applicable regulatory or accreditation standards (e.g., ISO 8655).

21. Prepare the pooled QC mixture by combining equal-sized aliquots from every study sample in the project, as calculated in step 12, using the container labeled in step 17.

***Note:*** Ideally, the pooled QC should represent the full cohort. Keep the pooled QC mixture on ice while aliquoting multiple batches of samples.

22. Immediately return the original sample tubes and remaining aliquots to −80°C.

***Note:*** Do not allow samples to remain at ≥ 4°C longer than necessary, and minimize exposure to light, oxygen, and heat.

23. Repeat steps 18–22 for subsequent randomized batches of 10 samples until all study samples have been aliquoted.
24. After preparing the pooled QC mixture from small amounts of all samples, vortex for 30 seconds to ensure homogeneity.
25. Aliquot 5 μL of the pooled QC into pre-labeled microcentrifuge tubes.

***Note:*** Prepare, at a minimum, one QC aliquot per extraction batch, plus three initial QC replicates. Prepare additional QC aliquots to allow for potential reinjection or re-extraction. Use the same type, manufacturer, and lot number of colorless microcentrifuge tubes for all samples and QC aliquots.

**Pause point:** Aliquoted serum samples can be stored at −80°C for up to one year.

### Lipid extraction

**Timing: 2 h per batch of 12 extractions (10 samples, one QC and one blank)**

**Figure 2:**
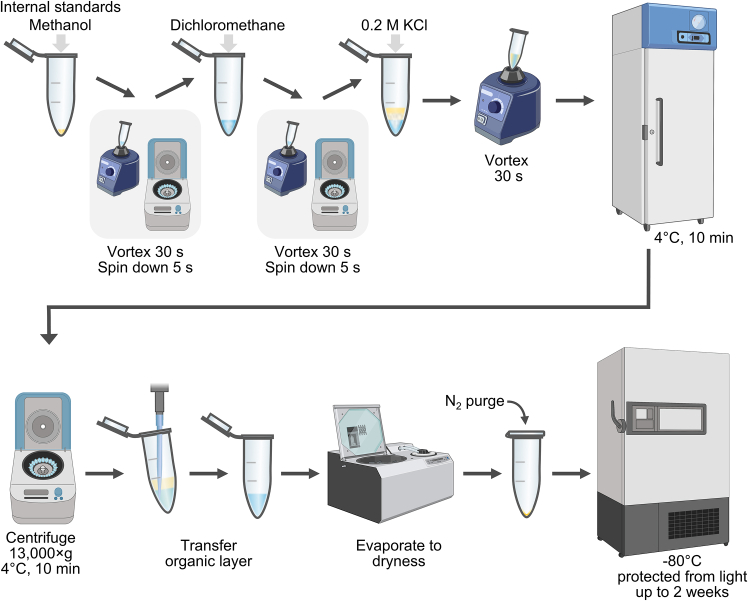
Lipid extraction workflow Serum samples are mixed with methanol, dichloromethane, and 0.2 M KCl, with a 30 s vortex in between additions. Samples are incubated for 10 min before centrifugation to promote phase separation. An aliquot of the organic phase is collected and evaporated to dryness. The dried extract is purged with nitrogen and stored at −80˚C. Icons were obtained from BioRender.

Here, we describe steps for extracting lipids from serum using a biphasic liquid-liquid extraction based on the Folch protocol optimized for untargeted LC-MS lipidomics (Figure 2).^1^^,^^2^

**CRITICAL:** It is strongly recommended to practice the extraction protocol with test samples before applying it to valuable or limited biological material.

**CRITICAL:** This protocol uses plastic microcentrifuge tubes and pipette tips for practicality and throughput. However, organic solvents can extract plasticizers and polymer-derived contaminants, which generate background peaks overlapping with endogenous fatty acids and other lipids. Variability in leachables between manufacturers and lot numbers further increases analytical noise. To minimize contamination, use colorless, high-quality plastic consumables from a single manufacturer and lot number for the entire project; minimize contact time between organic solvents (including their vapors) and plastic materials; do not allow methanol or 2-propanol to contact polysiloxane surfaces or non-PTFE vial caps; prepare and store organic solvents, mobile phases, and calibration solutions exclusively in glass containers with PTFE-lined caps and replace often; and maintain consistent timing across samples during extractions to standardize solvent–plastic exposure.

**CRITICAL:** Environmental contaminants are readily detected by untargeted LC–MS. To prevent contamination, clean benches and surfaces with 70% ethanol or 2-propanol before and after use; keep solvent bottles tightly closed; avoid working over open sample tubes; and avoid talking or unnecessary movement over open containers.

**CRITICAL:** Prepare samples following a fully randomized sequence (independent of sample grouping or attributes), as defined in the Project design and experimental planning section. The extraction must be performed quickly but carefully. Keep solvent bottles open only for the minimum time required to transfer the solvent to minimize volatilization and contamination. Organic solvents and extracts should not be stored in plastic tubes, even for short periods (see troubleshooting – problem 2). Proceed through the extraction workflow without interruption until the solvent is evaporated to dryness.

**CRITICAL:** Volatile organic solvents can drip rapidly from pipette tips, leading to inaccurate volumes (see troubleshooting – problem 3). Pre-wetting the tip before transfer is essential. For dichloromethane, rinse the pipette tip with the solvent at least six times prior to dispensing the measured volume.

**CRITICAL:** Handle solvents while wearing appropriate PPE, including a laboratory coat, non-powdered nitrile gloves, and safety glasses. Ensure adequate laboratory ventilation or perform solvent handling in a certified chemical fume hood.

***Note:*** Prior to extraction, transfer small working volumes of solvents (5–10 mL of methanol, dichloromethane, water, 0.2 M KCl solution, MPA, and MPB) into clean glass bottles with PTFE-lined caps, previously rinsed with the same solvent or solution. Replace the solvent aliquots daily and discard any unused solvent at the end of the day to minimize contamination.

26. Pre-cool a refrigerated centrifuge to 4°C. Prepare all materials before starting.
27. Remove one ampule of the SPLASH internal standard mixture from −20°C.

**CRITICAL:** Add a defined mixture of deuterated lipid internal standards (SPLASH Lipidomix Mass Spec Standard, Avanti Research) to each sample prior to extraction to monitor extraction efficiency and correct for analytical variation, including ion suppression and transmission effects. The inclusion of an internal standard mixture is essential for reliable normalization and quantitative comparison across samples.

28. Vortex the ampule for 60 s at 20°C–24°C.
29. Carefully break the ampule open and transfer the contents into clean, dry amber glass vials with 300 μL fixed glass inserts, previously rinsed with LC-MS-grade methanol. Divide the solution into aliquots sufficient for one day of extractions.
30. Tightly seal the vials containing the SPLASH internal standard mixture with PTFE-lined caps.
31. Store the internal standard aliquots at −20°C until use.

***Note:*** The Avanti SPLASH Lipidomix mixture used in this workflow is supplied in methanol. Do not allow the solution to contact vial caps or liners, as these can leach contaminants into organic solvents. Avoid prolonged exposure to air to prevent solvent evaporation and concentration drift. Keep vials tightly capped when not in use, minimize open time during pipetting, and avoid repeated freeze–thaw cycles of the standard mixture. Evaporation of solvent will alter internal standard concentrations and compromise quantitative normalization (see troubleshooting – problem 3).

32. Prepare one blank aliquot by transferring 5 μL of water to a labeled microcentrifuge tube. Use the same tube type and size, manufacturer, and lot number as for the sample and QC aliquots.
33. Remove one vial of the SPLASH internal standard mixture from −20°C and vortex for 30 s at 20°C–24°C.
34. Remove ten sample aliquots (following the randomized order) and one QC aliquot from −80°C.

**CRITICAL:** Do not allow the samples to thaw before proceeding.

35. Add 4 μL of SPLASH internal standard mixture to each sample, QC aliquot, and blank using a new, pre-wetted pipette tip for each transfer.
36. Add 163 μL methanol to each sample, QC, and blank using a new, pre-wetted pipette tip for each transfer.
37. Tighlty close the tubes, vortex 30 s, and spin down for 5 s using a mini-centrifuge.

***Note:*** Methanol addition causes protein precipitation, producing white or cream-colored particles.

38. Add 333 μL dichloromethane using a new, pre-wetted pipette tip for each transfer.

***Note:*** Dichloromethane drips rapidly from pipette tips. Pre-wet pipette tips by pipetting and dispensing the solvent 6 times before aliquoting the required volume to improve reproducibility (see troubleshooting – problem 3). Dichloromethane is highly volatile; minimize exposure to air and avoid prolonged contact with plastic materials. Perform each step consistently across samples to ensure reproducibility.

39. Vortex 30 s and spin down for 5 s using a mini-centrifuge.
40. Add 120 μL of 0.2 M potassium chloride solution using a new, pre-wet pipette tip for each transfer (final ratio of 8:4:3 dichloromethane/methanol/aqueous).
41. Vortex for 30 s.

***Note:*** The aqueous solution promotes the formation of a biphasic system consisting of an aqueous upper layer, an organic lower layer, and a thin protein pellet at the interface.

42. Incubate samples, QC, and the blank extract at 4°C for 10 min, protected from light.
43. Centrifuge at 13,000 × *g* at 4°C for 10 min.
44. Transfer 300 μL of the lower organic phase to a clean, labeled tube using a new, pre-wetted pipette tip for each transfer.
  a. Rinse a new pipette tip with cold dichloromethane at least six times.
  b. Insert the tip into the aqueous layer while touching the inner wall of the tube.
  c. Slide the tip along the wall through the aqueous layer and the protein pellet until it reaches the bottom organic layer.
    ***Note:*** Slightly tilting the tube may help position the tip against the wall, but do not tilt the pipette.
  d. Aspirate the organic phase using a controlled release of the pipette plunger. Do not allow the plunger to snap back, as this can cause bubbles and loss of reproducibility.
  e. Hold the pipette tip in the organic phase for ≈1 s to allow complete filling.
  f. Transfer immediately into a new, pre-labeled tube.
    **CRITICAL:** Dichloromethane drips quickly from the pipette tip; perform the transfer quickly, consistently, and without delay to improve reproducibility (see troubleshooting – problem 3). Do not transfer protein material or aqueous phase into the organic extract.
    ***Note:*** A small amount of organic phase remains behind in the original tube after transferring the volume specified in step 44 to prevent disturbance of the protein pellet. If particulate material is accidentally aspirated, return the liquid to the original tube, minimizing disturbance of the layers, and centrifuge again for 5 min at 13,000 × g and 4°C.
45. Evaporate organic extracts to dryness at 20°C–24°C using either a nitrogen blow-down evaporator (≈15 min) or a vacuum concentrator (≈30–45 min).

**CRITICAL:** Ensure no liquid remains after drying. Monitor frequently and cap tubes immediately once the solvent has evaporated. Do not dry samples longer than necessary.

46. Purge dried extracts with nitrogen, immediately cap the tubes, and store at −80°C protected from light.

**CRITICAL:** Once lipid extraction is initiated, proceed through all steps without interruption until samples are fully dried. Pauses between steps may introduce variability due to differential solvent exposure, evaporation, or lipid degradation and aggregation.

**Pause point:** Dried lipid extracts may be stored at −80°C, under nitrogen and protected from light, for up to 2 weeks. However, lipid extracts are prone to degradation during storage; samples should be prepared as close to LC–MS analysis as possible.

### Resuspension of lipid extracts for LC-MS analysis

**Timing: 45 min per batch of 10 samples, one QC and one blank extract**

**Figure 3:**
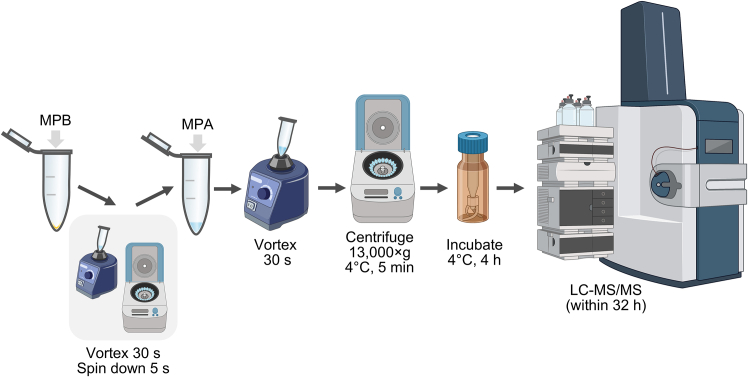
Extract resuspension Dried extracts are resuspended in chromatographic mobile phases for LC-MS/MS analysis. Icons were obtained from BioRender.

Here, we describe steps to resuspend dried lipid extracts in chromatographic mobile phases for reversed-phase LC–MS analysis (Figure 3).

***Note:*** Dried lipid extracts dissolve more efficiently in a high-organic solvent medium; therefore, they are first resuspended in MPB. The extract is subsequently diluted with MPA to obtain a final 1:1 MPA/MPB composition. Resuspension is performed at 20°C–24°C using vortex mixing only; sonication is not recommended, as it may promote lipid degradation. Under these conditions (5 μL serum extract resuspended in 50 μL of 1:1 MPA/MPB), lipid films dissolve readily, and complete resuspension is consistently achieved without additional intervention. Although dichloromethane or chloroform are commonly used to solubilize lipid extracts, these solvents are poorly compatible with reversed-phase LC gradients containing aqueous solvents and may cause phase separation, lipid precipitation, peak distortion, and potential clogging of the column inlet. This workflow uses mobile phases rather than dichloromethane for greater robustness and analytical performance.

**CRITICAL:** Samples should be kept at 4°C immediately after resuspension until analysis. Resuspend samples following the same randomized batch order used during extraction (no more than two batches of 10 samples, one QC, and one blank per day).

47. Transfer small working volumes (≈5–10 mL) of each freshly prepared mobile phase to clean glass bottles with PTFE-lined caps.

**CRITICAL:** Use freshly prepared mobile phases for resuspension (within 7 days). Mobile phases are volatile; keep flasks and resuspended sample vials tightly closed.

48. Remove dried lipid extracts from −80°C storage.
49. Add 25 μL of mobile phase B to each dried extract using a new, pre-wetted pipette tip for each transfer.
50. Vortex 30 s and spin down for ≈5 s using a mini-centrifuge.
51. Add 25 μL of mobile phase A and vortex for an additional 30 s.
52. Centrifuge at 13,000 × *g* at 4°C for 5 min.
53. Transfer the resuspended extract to labelled 2 mL screw-cap amber autosampler vials with disposable 250 μL inserts, immediately closing the vials with PTFE/silicone caps.

**CRITICAL:** Vial inserts have a conical bottom, which can trap air bubbles during transfer. Air bubbles within the vial insert can affect the injected volume and reduce reproducibility during LC-MS analysis. Inspect each insert for residual bubbles, particularly near the conical tip and spring feet, prior to analysis.

54. Vortex each vial for ≈5 s to remove trapped air bubbles, which can affect injection reproducibility in inserts with conical bottoms.
55. Firmly tap each vial on a hard surface and flick to further dislodge trapped air bubbles.

**Pause point:** Allow resuspended extracts to equilibrate at 4°C for 4 h prior to injection.

**CRITICAL:** Inject resuspended lipid extracts between 4 and 32 h after resuspension to minimize lipid degradation, aggregation, and adsorption losses.

### LC-MS/MS analyses

**Timing: 10 h per batch**

This section describes the LC–MS injection routine, following the parameters described in the materials and equipment section.

***Note:*** Reversed-phase LC separates lipids primarily according to hydrophobicity and fatty acyl composition. The combination of methanol, acetonitrile, and water in MPA provides enhanced selectivity through hydrogen bonding and π-interactions, while 2-propanol in MPB promotes elution of highly hydrophobic lipids. Ammonium formate is included in both mobile phases to facilitate adduct formation and improve detection across lipid classes. Extracts are analyzed in positive- and negative-ionization modes to maximize lipid coverage.

**CRITICAL:** Accurate mobile phase composition is critical, as small deviations can cause retention-time shifts and altered ionization.

56. Load both mobile phases into the LC lines and purge the system with 50% MPB for 5 min at 3 mL/min.
57. Stabilize the LC–MS system with 10% MPB while maintaining the column oven at 50°C for 30 min.

***Note:*** The column oven is maintained at 50°C to reduce solvent viscosity and improve chromatographic performance with isopropanol-rich mobile phases.

58. Verify system performance by monitoring LC backpressure and baseline signal intensity for 15 min at each of the following conditions: 10% MPB, 0.350 mL/min (≈400–550 bar); 50% MPB, 0.350 mL/min (≈600–750 bar); and 95% MPB, 0.280 mL/min (≈750–900 bar).

**CRITICAL:** Confirm that system pressure is stable (variation ≤ ±1% for at least 15 min) and below 1,000 bar under each condition described in step 58 before proceeding. Assess baseline intensity under the same conditions; variation should remain within ±30% and below 5 × 10^4^ counts (base peak chromatogram) over a minimum of 15 min.

59. Equilibrate the system at 10% MPB, 0.350 mL/min, and 50°C for 15 min before starting the injection sequence.

**CRITICAL:** Ensure the column is fully equilibrated at 10% MPB, 0.350 mL/min, and 50°C before starting the injection sequence. Stable backpressure (variation ≤ ±1%) and baseline signal (variation within ±30% and below 5 × 10^4^ counts for base peak chromatogram) should be observed for at least 15 min prior to injection.

***Note:*** Lipids can accumulate on column frits and LC tubing over time. Monitor system backpressure throughout the sequence and flush the LC system periodically with 100% 2-propanol to prevent buildup (see troubleshooting – problem 4).

**CRITICAL:** Track chromatographic performance throughout the project by monitoring peak shape, retention-time stability, and system pressure using pooled QC samples. Progressive peak broadening or loss of peak symmetry typically indicates column aging (see troubleshooting – problem 3). Sudden pressure changes or retention time drift may indicate particulates in mobile phases, column contamination, precipitation at the column inlet or injection system, or mobile-phase instability (see troubleshooting – problem 4). Under the conditions described here, reversed-phase columns may support extended use (in some cases exceeding 6,000 injections); however, column lifetime is highly dependent on operating conditions, and chromatographic performance should be periodically assessed.

60. Perform mass and ion mobility (CCS) calibration of the mass spectrometer in positive- and negative-ionization prior to sample analysis.
  a. Open the positive ionization method.
  b. Set the HESI Sheath Gas Temperature to 50°C.
  c. Wait 15 min for instrument equilibration.
  d. Infuse a 2:1 (v/v) mixture of Agilent ESI-L LC-MS Tuning Solution and 10 mM sodium formate solution via syringe pump at 50 μL/min directly to the ion source.
  e. Calibrate mass and CCS using Bruker timsControl.
  f. Record the calibrant signal intensity and the standard deviation of mass and CCS.
  g. Open the negative ionization method.
  h. Repeat steps b-f.
  i. Open the positive ionization method and wait 15 min for instrument equilibration before proceeding with injections.

**CRITICAL:** Ensure a calibration quality score of 100% and standard deviations ≤0.50 ppm.

61. Infuse the 2:1 (v/v) mixture of Agilent ESI-L LC-MS Tuning Solution and 10 mM sodium formate solution via syringe pump at 40 μL/h through the 6-valve port, directed to the waste line, before starting injections.
62. Prepare the LC–MS injection sequence, using a 5 μL injection volume for positive ionization.
  a. 1–3 solvent blanks (1:1 MPB/MPA; see troubleshooting – problem 2).
  b. First extraction blank.
  c. First pooled QC.
  d. First batch of samples in a randomized order.
  e. Second extraction blank.
  f. Second pooled QC.
  g. Second batch of samples in a randomized order.
  h. One solvent blank.
63. Repeat step 62 for negative ionization, using 10 μL injections. Start the negative injections with one blank injection to allow for instrument equilibration.
64. Finish the sequence with at least one solvent blank (1:1 MPB/MPA).

***Note:*** Two batches of 10 samples each, one QC, and one blank typically require ≈20 h. Solvent blank injections are required to prepare the system and identify background contamination originating from solvents or carryover. Maintain consistent injection volumes (5 μL for positive, 10 μL for negative ionization), autosampler temperature (4°C–8°C), and needle-wash conditions throughout each study. A larger injection volume is used in negative ionization mode to improve detection of low-abundance acidic lipid classes (e.g., phosphatidylinositols, phosphatidic acids, phosphatidylserines, and cardiolipins), which typically produce weaker signals than those detected in positive mode.

**CRITICAL:** Do not perform more than three injections from the same vial (i.e., no more than three septum punctures per vial, across both ionization modes). Repeated septum punctures can release polysiloxane contaminants from the silicone liner, potentially interfering with lipid analysis. Transferring the sample to a new vial does not mitigate this issue, as contamination may already be introduced during prior injections. Prepare separate vials for each injection set when multiple analyses are required.

**CRITICAL:** Do not reuse autosampler vials containing resuspended lipid extracts more than 32 h after resuspension. Lipid degradation, solvent evaporation, and adsorption can alter sample composition. Discard any remaining extract and prepare fresh samples if reinjection is required.

### Post-acquisition quality control and run assessment

**Timing: 1 h per batch**

This section describes steps for detecting analytical issues and preventing the propagation of compromised data into downstream processing.

**Figure 4:**
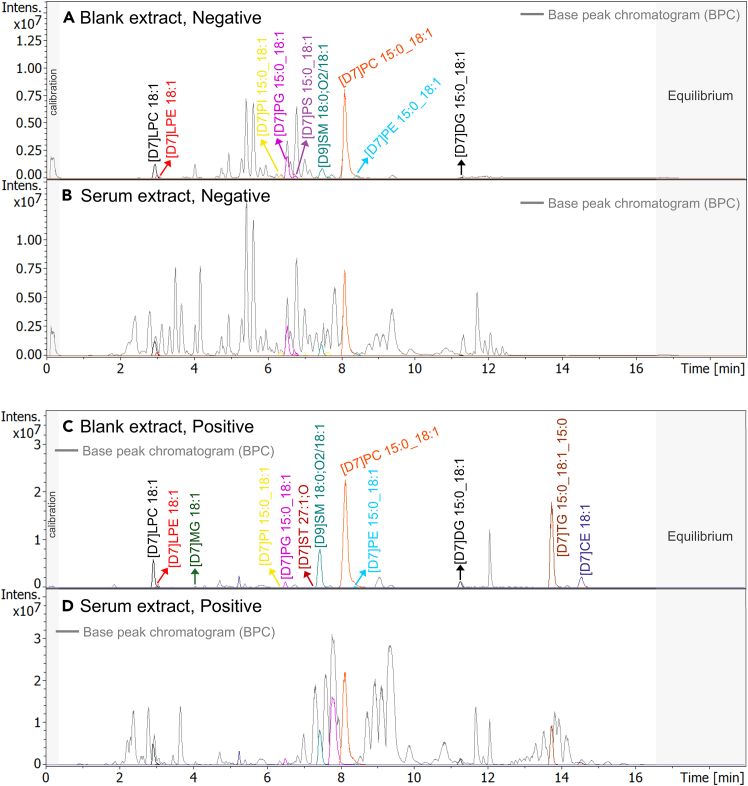
Chromatogram examples Examples of chromatograms obtained for (A) a blank extract in negative ionization (10 μL injection), composed of 5 μL of water and the internal standard mixture, which underwent all sample preparation steps; (B) human serum with internal standards in negative ionization (10 μL injection); (C) a blank extract with internal standards in positive ionization (5 μL injection); (D) human serum with internal standards in positive ionization (5 μL injection). Extracted ion chromatograms (EIC) for internal standards are shown in varied colors, whereas the gray trace corresponds to the base peak chromatogram (BPC). Lipid nomenclature follows community guidelines, where “[D7]” or “[D9]” represent 7 or 9 deuterium atoms replacing hydrogens, followed by lipid class abbreviations and fatty acyl chain composition (number of carbons:number of double bonds), e.g., [D7]PC 15:0_18:1 corresponds to a phosphatidylcholine standard with seven deuterium atoms, a saturated fatty acyl chain with 15 carbons, and a fatty acyl chain with 18 carbons and one double bond. When stereospecific positions relative to the glycerol backbone (sn) cannot be confidently attributed, fatty acyl chains are separated by “_”; if sn positions are known, “/” is used (e.g., [D9]SM 18:0;O2/18:1 corresponds to a sphingomyelin standard with nine deuterium atoms, a d18:0 sphingoid base, and an 18:1 fatty acyl chain).

65. Open all acquired datasets on Bruker DataAnalysis as soon as possible after injection (ideally, within the 32 h window after resuspension).
66. Recalibrate injections prior to data review using the calibration segment (0.0–0.3 min) for:
  a. Mass accuracy using the sodium formate clusters.
  b. Ion mobility using tuning mix reference values.
67. Generate extracted ion chromatograms (EICs) for the internal standard ions to support rapid visualization of chromatographic performance (Figure 4; Table 3) (see troubleshooting – problem 3).

**Table 1:** LC gradient elution for untargeted lipidomics, with MPA: 10 mM ammonium formate in 40:35:25 methanol/acetonitrile/water (v/v/v) and MPB: 10 mM ammonium formate in 96:3:1 2-propanol/acetonitrile/water (v/v/v)

Time (min)	MPB (%)	Flow rate (mL/min)
0.00	10.00	0.050
0.10	10.00	0.350
4.00	45.00	0.350
4.60	45.00	0.350
9.80	48.00	0.350
10.80	76.00	0.350
15.20	85.00	0.320
15.50	99.00	0.300
15.90	99.00	0.300
16.30	10.00	0.250
16.90	10.00	0.350
18.80	10.00	0.350
19.85	10.00	0.350
19.90	10.00	0.350
20.00	10.00	0.350

A reduced flow rate is used for high MPB proportions to maintain the system pressure within acceptable limits (≤1,000 bar). No sample peaks elute before 0.3 min (void volume) or after 16.5 min (column re-equilibration).

**Table 2:** MS method parameters for untargeted lipidomic analysis using TIMS-PASEF (total acquisition time: 20 min)

Acquisition segments		
0.0 – 0.3 min	Calibration: infuse a 2:1 (v/v) mixture of Agilent ESI-L LC-MS Tuning Solution and 10 mM sodium formate solution via a syringe pump set to 40 μL/h through a 6-port diverter valve (no PASEF acquisition; scan mode: MS). The divert valve directs flow to waste for 0.3 min during the initial calibration window, then switches to the mass spectrometer for the remainder of the run.	
0.3 – 20.0 min	Run (TIMS-PASEF acquisition; scan mode: PASEF)	
Source Parameters		
Source Type	VIP-HESI	
End Plate Offset	500 V	
Capillary	4300 V	
Nebulizer	2.0 bar	
Dry Gas	8.0 L/min	
Dry Temperature	220°C	
Sheath Gas Temperature	300°C	
Sheath Gas Flow	3.0 L/min	
TIMS Settings	Positive ionization	Negative ionization
1/K0 Start	0.60 V. s/cm^2^	0.60 V. s/cm^2^
1/K0 End	2.00 V. s/cm^2^	1.98 V. s/cm^2^
Ramp Time	65.0 ms	65.0 ms
Accumulation Time	65.0 ms	65.0 ms
Duty Cycle	100.00%	100.00%
Ramp Rate	14.05 Hz	14.05 Hz
Tune	Positive ionization	Negative ionization
Deflection 1 Delta	70.0 V	−70.0 V
Funnel 1 RF	500 Vpp	500 Vpp
Funnel 2 RF	250.0 Vpp	250.0 Vpp
isCID Energy	0.0 eV	0.0 eV
Multipole RF	200.0 Vpp	200.0 Vpp
Collision Energy	6.0 eV	6.0 eV
Collision RF	1450.0 Vpp	1450.0 Vpp
Quadrupole Ion Energy	5.0 eV	5.0 eV
Quadrupole Low Mass	150.00 m/z	150.00 m/z
Transfer time	70.0 μs	70.0 μs
Pre Pulse Storage	5.0 μs	5.0 μs
Δt1 (Deflection Transfer -> Capillary Exit)	−20.0 V	20.0 V
Δt2 (Deflection Transfer -> Deflection Discharge)	−120.0 V	120.0 V
Δt3 (Funnel 1 In -> Deflection Transfer)	80.0 V	−80.0 V
Δt4 (Accumulation Trap -> Funnel 1 In)	90.0 V	−100.0 V
Δt5 (Accumulation Exit -> Accumulation Transfer)	0.0 V	0.0 V
Δt6 (Ramp Start-> Accumulation Exit)	140.0 V	−140.0 V
Collision Cell In	200.0 V	−200.0 V
Ion Charge Control	7.00 M	7.00 M
Mass Spectra Peak Detection – Absolute threshold	100	100
Mobility Peak Detection – Absolute threshold	100000	100000
PASEF Data Reduction – Denoising mode	No denoising	No denoising
MS/MS Acquisition	Positive ionization	Negative ionization
Scan mode	MS between 0 and 0.3 min; PASEF between 0.3 and 20.0 min	
m/z range	120 to 1850	120 to 1850
Number of PASEF ramps	2	2
Total cycle time	0.36 s	0.36 s
Charge Minimum	0	0
Charge Maximum	1	1
Scheduling	Simple, target intensity 8000, intensity threshold 200	
Active exclusion (enabled), release after	0.08 min	0.08 min
Collision energy (collision-induced dissociation, CID; scan #1)	22 to 28 eV (1/K_0_ 0.60 V·s/cm)	21 to 33 eV (1/K_0_ 0.60 V·s/cm)
Collision energy (CID, scan #2)	28 to 37 eV 1/K_0_ 2.00 V·s/cm)	27 to 39 eV (1/K_0_ 2.00 V·s/cm)
MS/MS stepping	Enabled	Enabled
Collision RF (scan #1)	1250 V	1250 V
Transfer time (scan #1)	65 μs	65 μs
Collision RF (scan #2)	1450 V	1450 V
Transfer time (scan #1)	70 μs	70 μs
Pre-pulse storage	5 μs	5 μs
Reference List for Calibration	[ESI] Na Formate for m/z; [ESI] Tunning Mix ED-TOF CCS compendium (ESI) for ion mobility collision cross sections (CCS)

**Table 3:** LC-MS parameters for deuterated lipid standards included in the SPLASH Lipidomix Mass Spec Standard (Avanti Research)

Standard	Formula	Expected retention time (min)	Positive ionization			Negative ionization		
			Most intense adduct	CCS	m/z	Most intense adduct	CCS	m/z
[D7] LPC 18:1	C_26_H_45_D_7_NO_7_P	2.6–3.0	[M+H]^+^	235.7	529.39935	[M+HCOO]^-^	243.4	573.39028
[D7] LPE 18:1	C_23_H_39_D_7_NO_7_P	2.8–3.0	[M+H]^+^	221.2	487.35240	[M-H]^-^	217.7	485.33785
[D7] MG 18:1	C_21_H_33_D_7_O_4_	3.5–4.0	[M+Na]^+^	197.1	386.32582	Not detected		
[D7] PI 15:0_18:1	C_42_H_72_D_7_O_13_P	6.2–6.7	[M+NH4]^+^	299.9	847.60359	[M-H]^-^	287.7	828.56249
[D7] PG 15:0_18:1	C_39_H_68_D_7_O_10_P	7.4–8.3	[M+NH4]^+^	290.3	759.58755	[M-H]^-^	273.5	740.54645
[D7] PS 15:0_18:1	C_39_H_67_D_7_NO_10_P	5.9–6.8	[M+H]^+^	282.8	755.55625	[M-H]^-^	277.5	753.54170
[D7] ST 27:1;O	C_27_H_39_D_7_O	7.1–7.8	[M+H-H2O]^+^	206.0	376.39549	Not detected		
[D7] PA 15:0_18:1	C_36_H_62_D_7_O_8_P	6.9–8.2	[M+NH4]^+^	272.4	685.55077	[M-H]^-^	258.3	666.50967
[D9]SM 18:0;O2/18:1	C_41_H_72_D_9_N_2_O_6_P	7.2–7.7	[M+H]^+^	292.4	738.64699	[M+HCOO]^-^	291.6	782.63792
[D7] PC 15:0_18:1	C_41_H_73_D_7_NO_8_P	7.8–8.6	[M+H]^+^	290.4	753.61337	[M+HCOO]^-^	291.2	797.60430
[D7] PE 15:0_18:1	C_38_H_67_D_7_NO_8_P	8.1–8.7	[M+H]^+^	275.5	711.56642	[M-H]^-^	267.3	709.55187
[D7] DG 15:0_18:1	C_36_H_61_D_7_O_5_	11.1–11.6	[M+NH4]^+^	263.4	605.58444	[M+HCOO]^-^	262.8	632.54882
[D7]TG 15:0_18:1_15:0	C_51_H_89_D_7_O_6_	13.6–14.5	[M+NH4]^+^	316.4	829.79847	[M+HCOO]^-^	308.4	856.76285
[D7] CE 18:1	C_45_H_71_D_7_O_2_	14.3–14.8	[M+NH4]^+^	292.7	675.67794	Not detected		

Retention time windows are reported as ranges, as retention times in LC–MS are sensitive to system-specific factors, including dwell volume and instrument plumbing.
68. Inspect each chromatogram (sample, QC, and blank injections) for:
  a. Retention time shifts across (threshold: ≤ 5 s).
  b. Elevated background signal or abnormal baseline behavior (e.g., baseline intensities increasing by more than 30% or extra peaks; see troubleshooting – problem 2).
  c. Inconsistent internal standard peak intensities (threshold: relative standard deviation ≤20% for peak heights) (see troubleshooting – problem 3).
  d. Evidence of degradation (presence of multiple, intense peaks corresponding to free fatty acids, lysophospholipids, and diacylglycerols; see troubleshooting – problem 1).

***Note:*** If sample thresholds are not met, reinject the affected samples if they remain within the 4–32 h window after resuspension and if vial cap septa have not been perforated more than twice. If these criteria are not met, re-extract the affected samples prior to analysis.

69. Compare pooled QC injections across the project to assess:
  a. Retention time reproducibility (threshold: ≤ 5 s).
  b. Injection-to-injection and batch variability, with particular attention to internal standard intensities (threshold: relative standard deviation ≤20% for peak heights).

***Note:*** Internal standard peak intensities in QC injections should typically exhibit <20% relative standard deviation (RSD) across the sequence; higher variability may be observed across biological samples due to matrix effects. Retention time variation for internal standards should generally remain within ±5 s across all injections.

***Note:*** If QC injections fail to meet acceptance criteria, reinject the affected batch (samples, QC, and blank) to ensure consistency of the quality control assessment across the sequence, provided it is within the 4–32 h window after resuspension and that the vial cap septa have not been perforated more than twice. If these criteria are not met, re-extract the batch.

70. Review blank injections to identify:
  a. Persistent background peaks or abnormal baseline intensities (e.g., baseline increasing by more than 30%; see troubleshooting – problem 2).
  b. Carryover from preceding samples.
  c. Contaminants originating from solvents, consumables, or the LC–MS system.

**CRITICAL:** Batches exhibiting significant deviations in retention time, signal stability, or internal standard response should be flagged for further inspection. If substantial deviations in QC performance or baseline behavior are observed, do not proceed with data processing until the issue is identified and resolved. Re-inject if necessary and feasible within the defined extract-resuspension window of 32 h.

**Pause point:** Once data acquisition is complete, datasets can be stored without time constraints prior to processing. Discard leftover samples in accordance with institutional requirements.

### Data processing

**Timing: Hours to days, depending on dataset size**

**Figure 5:**
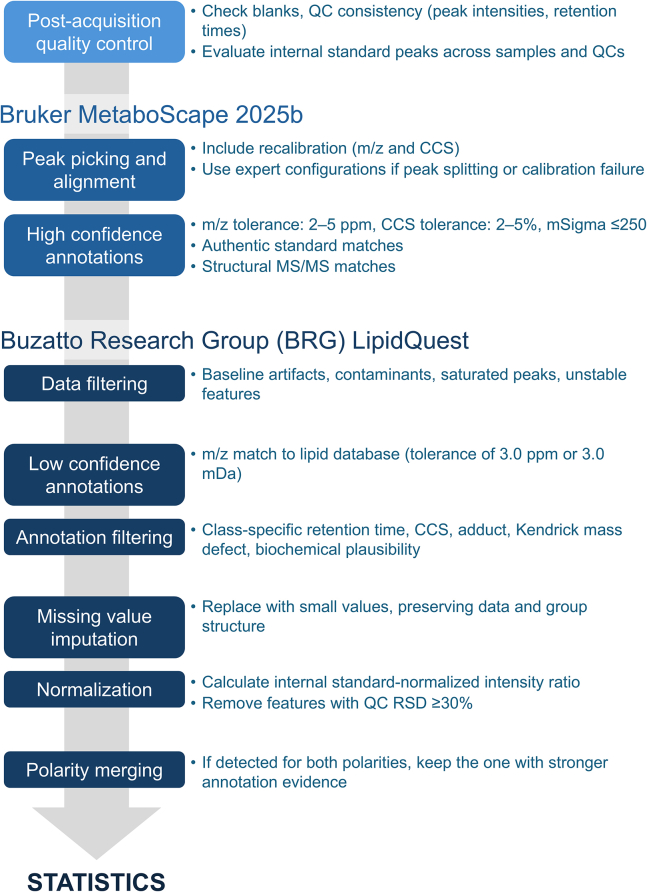
Data processing and quality control routine All acquired chromatograms are first visualized and inspected using Bruker DataAnalysis. Peak picking, alignment, and high-confidence annotation assignment are performed with Bruker MetaboScape. Downstream analysis uses custom Python-based scripts (suggested: BRG LipidQuest, https://github.com/Buzattoresearch/BRG-LipidQuest).

Here, we describe steps for post-acquisition processing of LC–MS data, including feature detection, alignment, and annotation (Figure 5).

***Note:*** Raw data are initially processed using Bruker MetaboScape 2025b for peak picking, retention time alignment, and ion deconvolution. The detected features are annotated at this stage through (i) target list matching to authentic standards based on retention time, accurate mass, collision cross section (CCS), and isotope pattern (mSigma), and (ii) MS/MS spectral matching supported by CCS information and isotope pattern (mSigma) (high confidence annotations). The processed data are subsequently exported and analyzed using the Buzatto Research Group LipidQuest pipeline (https://github.com/Buzattoresearch/BRG-LipidQuest, https://doi.org/10.5281/zenodo.19545764) to further refine the data. This includes removal of contaminants and low-quality features, additional mass matching (low confidence annotations), annotation filtering using analytical and biological plausibility criteria (including retention time behavior, Kendrick mass defect consistency, CCS trends, and class-specific constraints), consolidation of redundant features and polarities, and internal standard–based normalization.

71. Process positive- and negative-ionization datasets separately using Bruker MetaboScape 2025b.

**CRITICAL:** Do not merge positive- and negative-ionization datasets prior to feature detection, alignment, and annotation, as ionization polarity strongly influences adduct formation and feature detection.

72. Import raw LC–MS datasets into Bruker MetaboScape 2025b and verify correct file ordering, injection sequence, and sample group assignments.
73. Perform feature extraction and alignment. A detailed list of all data-processing parameters is provided in Table 4.
  a. Define the chromatographic window (0.3–16.5 min), excluding the calibration (0–0.3 min) and re-equilibration segments (16.5 – 20 min), during which no analytes elute.
  b. Apply mass recalibration with sodium formate clusters using the calibration segment (0.0–0.3 min).
  c. Apply ion mobility (CCS) recalibration using tuning mix reference values between 0.0 and 0.3 min.

**Table 5:** Optional expert configurations for MetaboScape 2025b

Filter Parameters	Value
FerraWorkflow.CalculateRtAlignment.alignIterations	4
FerraWorkflow.CalculateRtAlignment.debugTraceRtTol	20
FerraWorkflow.CalculateRtAlignment.lowessIterations	4
FerraWorkflow.CalculateRtAlignment.minNumPoints	10
FerraWorkflow.CalculateRtAlignment.rtDelta	30
FerraWorkflow.GroupFeatures.rtDelta	8
FerraWorkflow.ForeachAnalysis.FeatureFinder.Calibration. MassRecalibration.Calibration.intensityThreshold	1500
FerraWorkflow.ForeachAnalysis.FeatureFinder.Calibration. MassRecalibration.Calibration.minimalNumberOfMatchingSpectra	3
FerraWorkflow.ForeachAnalysis.FeatureFinder.Calibration. MassRecalibration.LockMassCalibration.minIntensity	1500

**Table 4:** Data processing parameters for MetaboScape 2025b

Filter Parameters		
Minimum # of features for extraction	Half of the number of injections in the smallest sample group (minimum 2). Example: if the smallest group contains 6 samples, set it to 3.	
Presence of features in the minimum # of analyses	Half of the number of injections in the smallest sample group (a minimum of 2)	
Filter features by occurrences in a group	Yes	
Presence of features in the sample group	80%	
T-ReX 4D Processing		
Intensity Threshold	5,000 counts	
Minimum 4D Peak Size	100 points	
Enable Recursive Feature Extraction	Yes	
Minimum 4D Peak Size (recursive)	70 points	
Retention Time Ranges	0.3 to 16.5 min	
Mass Range	120 to 1850 m/z	
Perform MS/MS import	Yes	
MS/MS import method	Average	
Group by collision energy	Yes	
Ion Deconvolution Parameters	Positive ionization	Negative ionization
EIC correlation	0.8	0.8
Primary ion	[M+H]^+^	[M-H]^-^
Seed Ions	[M+Na]^+^, [M+K]^+^, [M+NH_4_]^+^, [M+2Na-H]^+^, [2 M+H]^+^, [2 M+NH_4_]^+^	[M-Cl]^-^, [M+HCOOH-H]^-^, [M+Na+HCOOH-H]^-^, [M-CH_3_]^-^, [2 M-Cl]^-^, [2 M+HCOOH-H]^-^
Common Ions	[M+H-H_2_O]^+^	[M-H-H_2_O]^-^
Split Features, if potential isomers are detected	Yes	Yes
Recalibration	Positive ionization	Negative ionization
Lock Mass	338.341741 m/z	283.26425 m/z
Mass Recalibration	Sodium Formate from 0 to 0.3 min	
Mobility Recalibration	Tuning Mix from 0 to 0.3 min	

All parameters listed correspond to the configured settings used for feature detection and grouping in this workflow

***Note:*** Parameter values were optimized for untargeted lipidomics datasets acquired using the chromatographic and acquisition conditions described in this protocol, processed with Bruker MetaboScape 2025b. Peak intensities (heights) are used for downstream analysis rather than integrated peak areas. In untargeted lipidomics datasets with variable peak shapes and partial co-elution, peak heights often provide more robust quantitative estimates and are consistent with the default output of MetaboScape feature extraction.

***Note:*** The extended list of adducts included in Table 4 under *Ion Deconvolution Parameters* is used to support feature grouping during data processing and does not directly influence lipid annotation. Lipids ionize as multiple adduct species under the conditions used. In MetaboScape 2025b, these ion forms are merged based on co-elution and signal correlation to reduce redundancy. Lipid annotation is performed independently using accurate mass, MS/MS evidence, CCS, and retention time.

***Optional:*** Use Expert Configurations to define retention time and m/z tolerances if default values result in peak splitting or insufficient calibration performance (Table 5).

74. Once the dataset is processed, review mass calibration metrics (deviation threshold: ≤0.50 for mass and mobility), retention time stability (alignment deviation threshold: ≤5 s), and feature count consistency across injections.

***Note:*** For samples derived from the same organism and comparable biological conditions, feature counts should typically fall within ±20% across injections; larger deviations may reflect biological variability or analytical issues. Investigate any injections showing failed calibration, abnormal retention-time shifts, or extreme deviations in feature counts.

**CRITICAL:** Feature tables containing poorly calibrated or unstable injections must be investigated and reprocessed prior to downstream analysis.

75. Annotate all internal standard ions for positive- and negative-ionization modes using a target list containing their formulas, expected retention times, and collision cross sections (CCS) (Table 3). Apply an m/z tolerance of 2–5 mDa, retention time tolerance of 1.0 min, mSigma tolerance of 0–250, and CCS tolerance of 1.0%–3.0%.
76. Inspect boxplots of internal standard intensities across study sample groups and QC injections to assess analytical stability. Flag internal standard features exhibiting high variability (>30% across QC injections, missing values for sample injections), large mass errors (>5 mDa), retention-time deviations (>30 s), intensity drift, or peak splitting.

**CRITICAL:** If internal standard behavior indicates analytical instability, mass-to-charge measurement inaccuracy, or missing values, investigate the dataset before proceeding (see troubleshooting – problem 3).

77. Annotate aligned features using accurate mass, isotope pattern matching, ion mobility (CCS), and MS/MS spectral matching using Bruker MetaboScape 2025b, according to the thresholds defined in Table 6.
  a. Perform lipid annotation using the “Lipid Species Annotation” module in MetaboScape 2025b.
  b. Perform MS/MS spectral library searches using lipid-focused spectral libraries.

**Table 6:** Annotation parameters for Bruker MetaboScape 2025b (annotation tier: high confidence)

Parameter	Lipid Species Annotation	Spectral Library Annotation
Only allow annotations validated by MS/MS spectra	Yes	Yes
Attempt primary ion reassignment	Yes	N/A
m/z tolerance	2 to 5 mDa	2 to 5 mDa
mSigma	250	250
MS/MS score	≥500	≥400
CCS	2.0% to 5.0%	2.0% to 5.0%
Search mode	N/A	Hierarchical
Scoring Algorithm	N/A	Matching mass tolerance: 5 mDa
MS/MS Libraries (suggested)	N/A	MoNA LipidBlast 2022^3^ MoNA HMDB^4^^,^^5^ (MoNA: Mass Bank of North America, https://mona.fiehnlab.ucdavis.edu/)

***Note:*** MetaboScape 2025b is used for annotations based on MS/MS matches, target matches to authentic standards, and CCS values, as described in Table 6 and steps 75 and 77. Low-confidence annotations based on mass match are performed with a stand-alone, in-house software (starting from step 82).

***Note:*** Lipid annotations reported by this workflow generally correspond to the molecular species level or the summed species level (total number of carbons and double bonds), unless confirmed using authentic standards.^11^ The positions of double bonds and stereochemical configurations cannot be confidently assigned using this method and should not be reported without further investigation. Lipids and related molecules can share the same elemental composition and exhibit highly similar fragmentation patterns. These structural isomers may differ only subtly, for example, in double-bond position, acyl chain arrangement, or stereochemistry, and cannot be reliably distinguished using this workflow without specialized fragmentation or derivatization methods. Consequently, distinct compounds may be annotated as the same lipid species despite differing in structure and biological function.

***Note:*** Lipid annotations based on combined evidence from accurate mass, isotope pattern, retention time, ion mobility (CCS), and MS/MS spectral matching are considered putative. Confirm annotations using authentic lipid standards whenever possible before drawing biological conclusions. When multiple lipid candidates correspond to a single feature, prioritize assignments supported by MS/MS spectra and consistent with expected mobility and retention behavior. Final lipid annotations should be interpreted in accordance with accepted community standards, including the guidelines of the International Lipidomics Society and the Lipidomics Standards Initiative.^11^^,^^12^

78. Manually review all lipid annotations by comparing experimental MS/MS spectra with reference spectra or by identifying characteristic diagnostic fragments (e.g., headgroup fragments, fatty acyl fragments, and diagnostic neutral losses).

***Note:*** Theoretical MS/MS spectra typically contain multiple diagnostic ions. However, experimental spectra may contain interfering fragment ions from co-eluting, isobaric compounds, which should be considered during manual inspection (see troubleshooting– problem 5). Features that fail annotation-specific criteria and manual inspection should have their lipid annotations removed, but may be retained as unannotated features for subsequent mass matching (see step 83).

79. Copy the curated feature tables for positive- and negative-ionization datasets directly from Bruker MetaboScape and paste them into Excel for additional downstream data processing and filtering.

***Note:*** Copy feature tables from a custom MetaboScape table view configured to include the following columns: RT [min]; m/z meas.; M meas.; Ions; Internal Standard; CCS (Å^2^); Mob. 1/K0; ΔCCS [%]; QC RSD [%]; Samples RSD [%]; Boxplot; Flags; MS/MS; Name; Annotations; Molecular Formula; Annotation Source; Δm/z [mDa]; Δm/z [ppm]; MS/MS score; mSigma; ΔRT; AQ Details; Samples RSD/QC RSD; followed by all sample intensity columns. Paste the dataset directly into a blank Excel spreadsheet. Direct copy–paste preserves the table structure required for downstream filtering and statistical processing. Examples are provided in the Supplementary Materials (Tables S1; S2 for positive- and negative-ionization modes, respectively).

80. Remove features if the blank signal contributes substantially to the observed intensity (sample intensity/blank intensity < 3).

***Note:*** Further data processing and filtering can be performed using vendor software, public tools, or custom scripts (Figure 5). In this workflow, steps 81–95 were implemented using Python scripts developed by the Buzatto Research Group (University of Calgary), available at https://github.com/Buzattoresearch/BRG-LipidQuest (https://doi.org/10.5281/zenodo.19545764). The BRG LipidQuest routine automates these steps, generating files ready for statistical analysis together with intermediate diagnostic outputs.

81. Apply the following filtering criteria to detected features to ensure analytical reliability and remove artifacts:
  a. Persistent blank-associated contaminants: Remove features consistently detected in blanks. Common examples include erucamide, polysiloxanes, polymers such as polyethylene glycol (PEG), and compounds originating from personal care products.
  b. Low-intensity features: Remove features with intensities below 3000 counts.
  c. Baseline and saturation artifacts: Remove near-constant peaks across samples at the extreme ends of the intensity distribution. These include:
    i. low-intensity baseline signals (features in the lowest 20th percentile of intensity, exhibiting RSD < 5% across samples).
    ii. high-intensity saturated signals (features within the 99th percentile of intensity exhibiting RSD < 5% across samples).
  d. Persistent low-level m/z signals: Remove features with repeated m/z signals detected throughout the injection, with low, similar intensities.
  e. QC instability filtering: Remove features with RSD ≥50% across pooled QC injections (raw intensities). Apply a stability-based rescue rule: features with RSD <30% in at least one non-QC group containing ≥3 replicates are retained.
  f. Missing value filtering: Remove features not detected in ≥80% of injections within at least one sample group.
  g. Adduct redundancy removal: Remove multiple adduct forms corresponding to the same chromatographic peak. Retain the most intense ion with the least proportion of missing values.
82. Assign lipid classes to all confidently annotated features following community standards (International Lipidomics Society, Lipidomics Standards Initiative, LIPID MAPS).^11^^,^^12^^,^^13^^,^^14^
83. Perform accurate-mass matching for features lacking confident MS/MS-based lipid annotations using the LIPID MAPS database. Set the precursor mass tolerance to ≤3 mDa.

**Figure 6:**
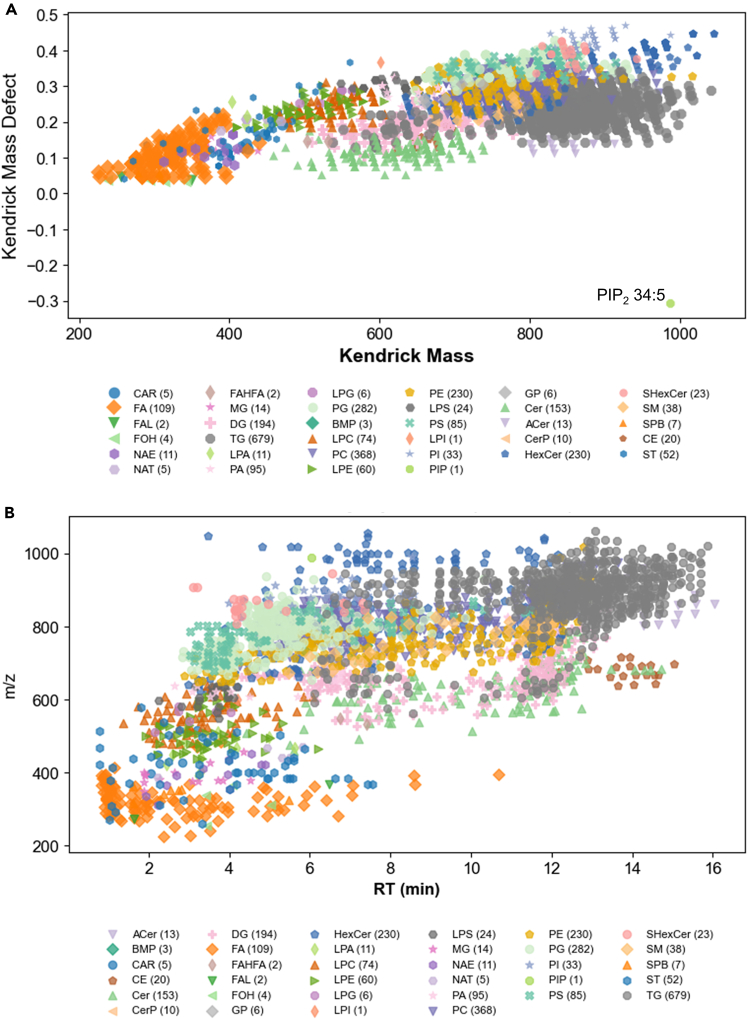
Quality control for lipid annotations, including high confidence (authentic standard and MS/MS + CCS matches) and low confidence assignments (mass matches) All annotations were filtered by expected analytical behaviour (retention time evaluation, class-specific Kendrick mass defect filtering, CCS trend filtering, adduct formation, removal of redundant or highly correlated features) and biochemical constraints (exclusion of non-mammalian lipid classes, implausible chain lengths or degrees of unsaturation, chemically unrealistic plasmalogens, and known contaminants), as described in the workflow. Each symbol/color combination corresponds to a different lipid class with varying fatty acyl compositions and functional modifications. All lipid annotations shown are putative and reflect the output of an untargeted annotation workflow; confidence varies across lipid classes and individual species, and assignments should be interpreted accordingly. Lipid annotations that do not follow class-specific behavior are potential misannotations and require further inspection. (A) Kendrick mass defect plot. The green dot labelled “PIP_2_ 34:5” exemplifies a potential misannotation that warrants further investigation. Although phosphatidylinositol phosphates (PIP) lipids are expected to show a shifted Kendrick mass defect because the additional phosphate groups break the CH_2_ homologous series, PIP_2_ are rare in serum, with mass-matches potentially arising from misassignment and requiring confirmation against authentic standards. (B) Mass-to-charge ratio (m/z) versus chromatographic retention times. Polar lipids have low retention in reversed-phase chromatography, thus eluting earlier (e.g., shorter-chain free fatty acyls, steroid lipids, lysoglycerophospholipids). Hydrophobic lipids have higher retention (e.g., very-long-chain glycerophospholipids, triacylglycerols, and esterified cholesterols). Lipid class abbreviations: CAR: acyl carnitines; FA: free fatty acids; FAG: fatty acyl glycosides; FAL: fatty aldehydes; FOH: fatty alcohols; HC: hydrocarbons; NA: fatty amines or amides; NAE: N-acyl ethanolamines and endocannabinoids; NAT: N-acyl taurines; WE: wax esters; FAHFA: fatty acid estolides (fatty acid esters of hydroxy fatty acids); MG: monoacylglycerols; DG: diacylglycerols; TG: triacylglycerols; LPA: lysophosphatidic acids; PA: phosphatidic acids; LPC: lysophosphatidylcholines; PC: phosphatidylcholines; LPE: lysophosphatidylethanolamines; PE: phosphatidylethanolamines; LPG: lysophosphatidylglycerols; PG: phosphatidylglycerols; LPI: lysophosphatidylinositols; PI: phosphatidylinositols; LPS: lysophosphatidylserines; PS: phosphatidylserines; BMP: bismonophosphates; PIP: phosphatidylinositol phosphates; CDP-DG: cytidine diphosphate diacylglycerols; Glc-GP: glycosylated glycerophospholipids; GP: other glycerophospholipids; Cer: ceramides; ACer: acyl ceramides; CerP: ceramide phosphates; GlcCer: glycosylated ceramides; HexCer: hexosylceramides; SCer: sulfoceramides; SHexCer: sulfatides; SM: sphingomyelins; LSM: lysosphingomyelins; SPB: sphingoid bases; HexSPB: hexosylsphingoid bases; SPBP: sphingoid base phosphates; SulfateHexSPB: sulfo-hexosyl sphingoid bases; CE: esterified sterols; ST: sterols and steroids.

**Figure 7:**
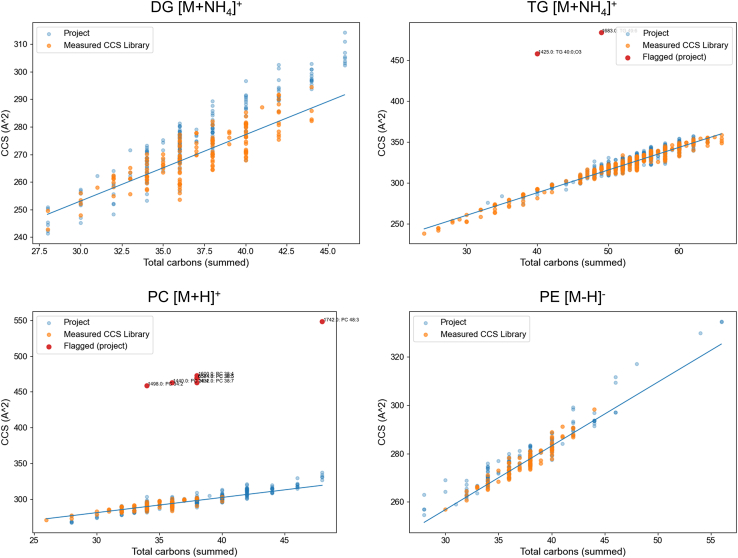
Examples of CCS distributions across lipids with different carbon numbers in their fatty acyl chains Annotations that do not follow class-specific behavior (shown as red dots) should be further investigated and, if not proven, removed. Investigate adducts individually, as their mobilities will differ substantially. Lipid class abbreviations are defined in Figure 6.
84. Apply the following orthogonal constraints to annotated lipids to reduce false-positive assignments (see troubleshooting – problem 5):
  a. Retention time constraints relative to annotated features of the same lipid class (Figure 6).
    ***Note:*** If authentic standards are available, restrict retention time within ±30 s of known values.
  b. Kendrick mass defect filtering within lipid classes to remove candidates whose mass defect is inconsistent with the expected class pattern (Figure 6).
  c. Expected adduct forms for each lipid class, determined empirically using authentic lipid standards analyzed under the same LC–MS conditions and acquisition settings.
    ***Note:*** Adduct patterns are used to prioritize biochemically plausible lipid annotations and to remove unlikely candidates.
  d. Ion mobility consistency, using CCS values relative to known lipid class and fatty acyl distributions (Figure 7).
  e. Biochemical plausibility, considering lipid classes and molecular species expected for the biological matrix under study.
    ***Note:*** Biochemical plausibility filtering restricts or removes implausible annotations to prevent propagation of biologically unsupported lipid assignments into downstream analysis.^15^^,^^16^ The BRG LipidQuest routine (https://github.com/Buzattoresearch/BRG-LipidQuest, https://doi.org/10.5281/zenodo.19545764) automatically applies the orthogonal filters described. The output of each filter can be investigated through debug files and plots generated as the data is processed. For example, mammalian serum samples typically do not contain ceramide phosphoinositols, phosphatidylinositol mannosides, galactosyl diacylglycerols, sulfoquinovosyl diacylglycerols, or saccharolipids. Fatty acyl chains with odd carbon numbers are detected at trace levels, but should not dominate lipid profiles. Carefully check the annotations to prevent misinterpretation within biological and biochemical constraints.
    **CRITICAL:** Tentative lipid annotations resulting from mass matching are considered provisional assignments (low confidence annotations). Mass or MS/MS matching does not constitute definitive lipid identification. Isomeric lipid species may remain ambiguously annotated or unresolved under untargeted acquisition conditions. Assignments should be interpreted with caution, and biological interpretation depends on the study question. Tentative annotations require targeted follow-up experiments and confirmation with authentic standards.
85. Resolve duplicate or redundant tentative annotations arising from:
  a. Multiple adduct forms corresponding to the same lipid molecule.
  b. Isotopologues or multimeric species.
  c. In-source fragment ions.
86. Impute missing intensities (non-detected signals). Replace them with small, biologically reasonable values derived from the dataset.
  a. For each lipid feature within each biological group, estimate a small reference value approximating the limit of detection (LOD) as the 1st percentile of positive intensities.
    ***Note:*** If this percentile cannot be computed due to insufficient positive values, apply a hierarchical fallback in the following order:
    i. The feature-specific 1st percentile across all samples and QCs.
    ii. The group-wide 1st percentile across all detected features within that group.
    iii. The dataset-wide 1st percentile across all positive intensities.
  b. If the feature is detected in ≥80% of injections within a group, replace missing values with one-third of the corresponding LOD estimate.
    ***Note:*** This assumes that non-detections in otherwise consistently detected features likely fall just below the detection limit.
  c. If the detection rate is intermediate (50%–80% of injections within the group), replace missing values with a conservative value defined as the larger of:
    i. One-third of the feature-specific LOD estimate, or.
    ii. One-fifth of the dataset-wide minimum intensity.
  d. If the feature is detected in <50% of injections within the group, replace missing values with one-fifth of the LOD estimate, reflecting greater uncertainty and avoiding artificial inflation of signal for sparsely detected features.
    ***Note:*** This imputation strategy is automatically applied by the BRG LipidQuest routine to preserve relative signal structure while minimizing artificial inflation of low-intensity features.
    ***Note:*** Missing values arise when low-abundance features fall below the detection limit in some injections, when signals are affected by matrix-dependent ion suppression, or when peak detection and alignment algorithms fail to consistently detect weak or abnormal chromatographic features. These non-detections do not necessarily indicate the absence of the lipid; rather, they reflect analytical and computational limitations. Many statistical procedures used in downstream analysis (e.g., fold-change calculation, multivariate modelling) cannot accommodate zeros or missing values; therefore, small intensity values that approximate the limit of detection are substituted to enable consistent statistical analysis while preserving the dataset and group structure.
87. Apply internal standard normalization.
  a. Internal standards are assigned to annotated features according to the following criteria:
    i. Class match: the internal standard belongs to the same lipid class as the annotated feature.
    ii. Structural similarity: if a lipid class does not have a matching internal standard, the internal standard that exhibits the most similar physicochemical properties and retention behavior within the chromatographic gradient is used (e.g., normalize Cer and HexCer, not represented in the SPLASH mixture, using the sphingomyelin (SM) internal standard).
    iii. Analytical performance: If the use of a class-matched internal standard increased QC variability by more than 20%, an alternative, structurally-similar internal standard providing improved reproducibility is selected. If no structural assignment is found, the internal standard providing the lowest QC variability across the dataset is selected. This approach is the only option for unannotated features.
  b. For each feature, calculate the intensity ratio: feature intensity (peak height)/internal standard intensity.

***Note:*** Lipid classes exhibit distinct ionization efficiencies, chromatographic retention behavior, and extraction recoveries. Using class-matched internal standards improves normalization by correcting for class-specific matrix effects and analytical variability. In this protocol, the SPLASH Lipidomix internal standard mixture (14 deuterated lipids representing 14 classes) is used for signal normalization and quality control. Normalization is performed using class-matched internal standards where available, and the closest appropriate internal standard within the same ionization mode otherwise (as described in Step 87). This approach supports relative comparisons but does not provide absolute concentrations.

**CRITICAL:** Use the same internal standard as the denominator for all features within the same lipid class and ionization polarity to maintain comparability across samples. If a selected internal standard significantly increases QC variability for a given class (>20%), the next most appropriate standard should be evaluated.

**CRITICAL:** Class-matched internal standards correct for class-dependent analytical variability but do not act as universal response factors. These normalized values do not represent absolute concentration. Ionization efficiency varies substantially with local matrix composition, lipid class, and molecular structure (e.g., chain length, unsaturation, and functional modifications). Class-matched normalization enables robust relative quantification, but absolute concentrations require calibration curves prepared using isotopically labelled authentic standards for each lipid species in the same biological matrix.

***Note:*** Median or total-signal (sum) normalization is sometimes applied in untargeted metabolomics workflows, but is generally not recommended for lipidomics datasets. These approaches assume that most detected features remain unchanged and that the total signal is comparable across injections. In lipidomics, this assumption is often violated because lipid metabolism frequently produces global shifts in lipid abundance or lipid-class composition, and results often contain outliers. Median or sum normalization can, therefore, artificially compress biologically meaningful differences or introduce systematic bias. Internal-standard normalization is preferred because it corrects extraction efficiency, matrix effects, ionization efficiency, and instrument response using chemically defined references. This approach preserves biologically meaningful variation in total lipid abundance and lipid-class distributions while controlling analytical variability.

88. Apply drift correction if required. Correct for gradual signal drift across the injection sequence using QC-based approaches such as locally estimated scatterplot smoothing (LOESS) or equivalent regression-based methods.
89. Assess feature stability using pooled QC injections. Remove features exhibiting QC relative standard deviation (RSD) ≥30% after normalization.

***Note:*** QC-based filtering ensures that only analytically reproducible signals are propagated to downstream statistical analyses.

90. Merge positive- and negative-ionization feature tables. After polarity-specific processing, quality filtering, and normalization, combine the positive- and negative-ionization results into a single table for downstream interpretation. Two acceptable approaches are:
  a. Simple merge (concatenation): Concatenate the curated positive and negative feature tables row-wise.
    ***Note:*** Retain a polarity identifier for each feature to preserve traceability. Unannotated features are always concatenated.
  b. Best-polarity merge for annotated features: Select a single “best” polarity per lipid feature when both polarities annotate the same underlying compound within a retention time tolerance of 12 s and a neutral mass tolerance of 5 ppm or 5 mDa.
    ***Note:*** Select the polarity with stronger annotation evidence (highest MS/MS score, lowest m/z error) and best reproducibility (lowest RSD for QCs). If no compatible match is found, retain the feature in its original polarity.
    ***Note:*** Polarity merging resolves redundancy and selects the more reliable representation of the same lipid signal when both polarities provide evidence. Step 90 is automatically applied by the BRG LipidQuest routine.
91. The obtained list of annotated peaks represents the output of this workflow; proceed with statistical analysis.

**Figure 8:**
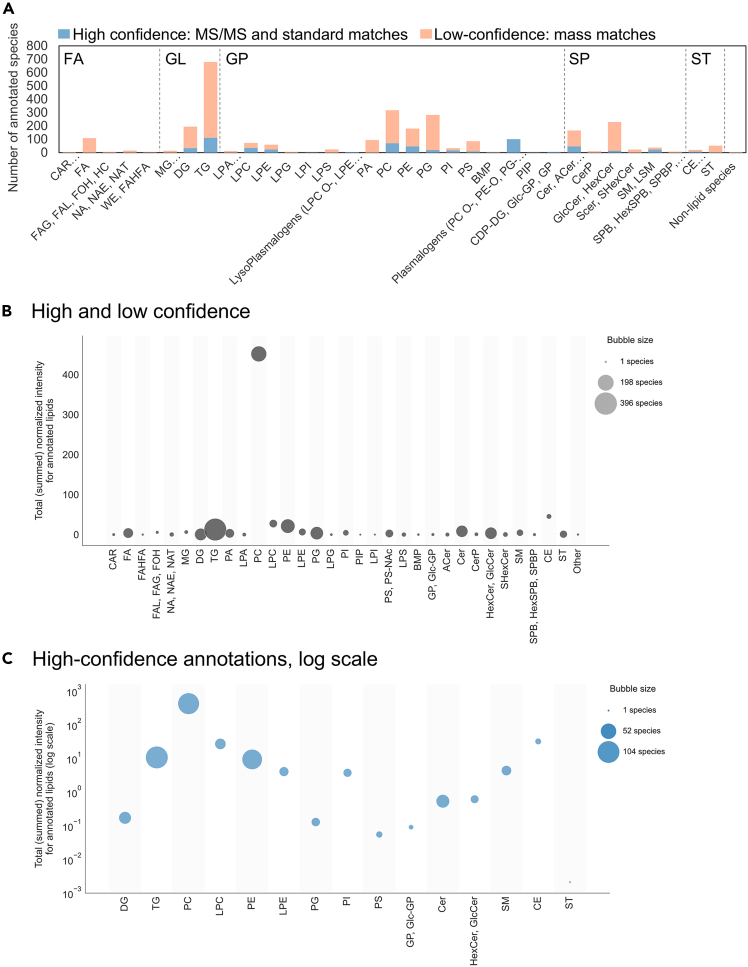
Distribution of lipid annotations obtained for human serum samples (Human Serum, Normal, Millipore-Sigma, Catalog No. S1-100ML; n = 3 experimental replicates) (A) Number of annotated lipid species per class. High confidence annotations correspond to MS/MS structural matches and authentic standard matches. Low confidence annotations are accurate mass matches (within 3.0 mDa and 3.0 ppm). Both confidence levels were submitted to retention time, adduct, CCS, Kendrick mass defect, and biochemical plausibility filtering. (B) Total (summed) normalized peak intensity distribution for high- and low-confidence annotations across lipid classes, with bubble sizes representing the number of different lipid molecules. (C) Total (summed) normalized peak intensity distribution restricted to high confidence annotations (log scale). Lipid class abbreviations are defined in Figure 6.

***Note:*** An example of the final output is provided as supplementary material (Table S3). This example dataset, obtained using commercial human serum samples, yielded 563 MS/MS-supported annotations (high confidence tier) and 2,288 mass-matched annotations (low confidence tier) (Figure 8). However, the final number of detected and annotated features is not a fixed performance metric and will vary depending on sample composition, study design, and analytical conditions. The values reported in this protocol reflect the example dataset used to demonstrate the workflow and should not be interpreted as a general expectation.

***Note:*** Low-abundance lipids such as hydroxycholecalciferol may be detected in untargeted datasets under the described conditions; however, their recovery and annotation depend strongly on sample composition and analytical sensitivity.

**CRITICAL:** Biological interpretation of lipid composition requires consideration of the organism, sample type, and study design, including expected biological alterations. Potential contributions from contaminants (e.g., solvents, plastics, or the laboratory environment) should also be evaluated. Blank filtering, defined as the exclusion of features detected in blank injections, is recommended to reduce background signals and improve annotation confidence. Thresholds may be adapted to the study; however, as a general guideline, features should be excluded prior to statistical analysis if their intensity in biological samples is less than threefold higher than that observed in blank injections.

**Pause point:** The processed dataset can be stored indefinitely prior to statistical analysis and biological interpretation.

### Statistical analysis

**Timing: Variable (hours to days, depending on dataset size)**

This section describes steps for the statistical testing and visualization of curated lipid feature tables after filtering, imputation, and internal-standard normalization.

***Note:*** A log transformation is neither required nor recommended for this workflow. Log transformation can amplify variability at the low-intensity end, particularly for sparsely detected features. Apply normality and homoscedasticity tests before proceeding, and use appropriate statistical models for non-normal datasets if needed.

***Note:*** Filter the obtained annotations by confidence tiers before downstream statistical analysis as required for each study (e.g., use only supported high-confidence annotations for biological interpretation).

**Figure 9:**
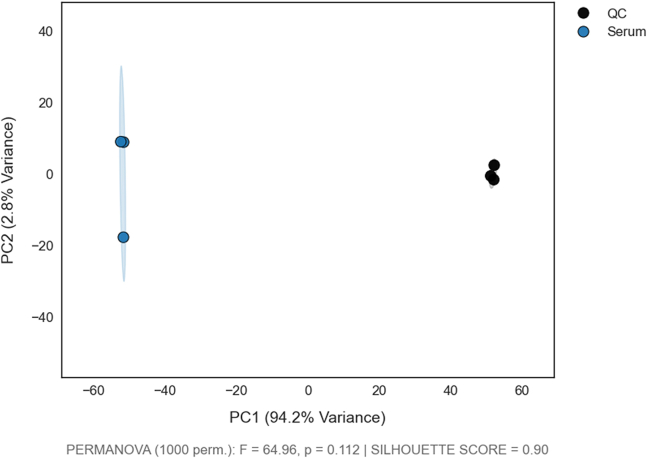
Example of a PCA scores plot used for data quality assessment, depicting tightly clustered QC replicates QCs are experimental replicates with the same biological composition and therefore show minimal variation. Apparent separation of the “Human Serum, Normal” replicates occurs along PC2, which explains only 2.8% of the total variance, indicating minor technical variability relative to the overall dataset structure. PERMANOVA (1000 permutations) F = 64.96, *p* = 0.1. Silhouette score = 0.9.

92. Investigate overall lipid class distribution using bar or bubble plots (Figure 8).
93. Perform Principal Component Analysis (PCA) to visualize global variance structure, clustering of QC experimental replicates, and identify outliers or batch effects (Figure 9).
  a. For PCA and other multivariate distance-based analyses, apply autoscaling (mean centering and unit-variance scaling) to prevent high-abundance lipids from dominating the variance structure.
  b. To quantify whether predefined sample groups differ in multivariate space, compute PERMANOVA on the full autoscaled feature matrix using an appropriate distance metric (typically Euclidean for autoscaled data).

**CRITICAL:** Autoscaling (mean-centering and variance scaling to unit standard deviation) is not applied for univariate testing of absolute intensity differences, but is required for variance-sensitive multivariate analyses (e.g., PCA, hierarchical clustering, and other distance-based methods) to prevent high-abundance lipids from dominating the analysis.

***Note:*** PCA provides an unsupervised, low-dimensional view of the major sources of variance in the dataset. In an unsupervised multivariate space (2-dimensional PCA scores plot), pooled QC injections should form a tight cluster that reflects analytical reproducibility. QC dispersion should be lower than dispersion among biological replicates within groups. If QC samples scatter broadly or drift along the injection sequence, treat the batch as analytically unstable and investigate before downstream statistics.

**CRITICAL:** PCA is commonly used to visualize analytical stability and clustering of QC samples. However, lipid species are highly correlated variables, and PCA can be difficult to interpret in high-dimensional lipidomics datasets. Use PCA primarily for quality control rather than biological interpretation. Biologically meaningful differences may exist even when groups overlap in the first two components. PCA loadings identify features contributing to variance along principal components, but large loadings do not imply biological importance and should not be interpreted as differential regulation without further statistical testing.

94. For datasets excluding QC injections, perform the following analyses as appropriate for the experimental design.
  a. Partial Least Squares – Discriminant Analysis (PLS-DA) for supervised separation of biological groups.
    i. Evaluate PLS-DA models using internal cross-validation and permutation testing before interpreting score separation or Variable Importance in the Projection (VIP) rankings.
    ii. Use 5-fold cross-validation to estimate predictive performance, and report R^2^ (apparent fit) and Q^2^ (predictive metric) values. Low Q^2^ values indicate an overfitted model.
    iii. Assess model significance using a label-permutation test. Randomly permute class labels (for example, 100 permutations), refit the model with the same number of components, and compare the observed model performance to the null distribution obtained under permuted labels.
      ***Note:*** A low permutation p-value indicates that model performance exceeds what is expected by chance given the dataset structure. A high permutation p-value indicates that observed separation can arise from chance correlations and should not be interpreted.
    iv. Interpret PLS-DA separation and VIP scores only when cross-validated Q^2^ and accuracy indicate predictive performance and when permutation testing supports significance.
  b. Heatmaps (clustered or not) to visualize group-wise abundance patterns and clustering behavior.
  c. Box plots and violin plots for visualization of selected features or lipid classes across groups.
  d. Correlation analysis to evaluate associations between lipid features and continuous metadata variables.
  e. Lipid class distribution plots and total intensities per class to summarize class-level shifts across biological groups.
    **CRITICAL:** QC injections are not biological replicates and must not be included in group comparisons, hypothesis testing, or supervised classification models, such as PLS-DA. QC and blank injections are excluded from all hypothesis testing by default; however, they are retained for quality assessment and filtering prior to downstream statistical analysis.
95. Perform Volcano analysis for pairwise group comparisons, including effect size and multiple-testing correction.
  a. Compute a two-sided p-value for each annotated lipid using either Welch’s two-sample t-test (parametric, unequal variance), or Mann–Whitney U test (non-parametric).
    i. Adjust p-values across features within each comparison using the Benjamini–Hochberg false discovery rate procedure.^17^
    ii. Report both raw p-values and FDR-adjusted p-values.
  b. Compute fold changes as the ratio of group medians using non-zero intensities.
  c. Classify features as significantly altered using a combined threshold requiring:
    i. Raw p-value < 0.05.
    ii. FDR-adjusted p-value < 0.05.
    iii. Absolute fold change ≥ 1.5 (equivalently |log2FC| ≥ log_2_(1.5)).

***Note:*** Many lipids share biosynthetic pathways, structural backbones, or fatty acyl pools, leading to strong correlations among features. Multiple-testing procedures assume independence or limited dependence among variables. Since lipidomics datasets often contain structured correlations, adjusted p-values should be interpreted cautiously and evaluated together with effect size, reproducibility, and biochemical context.

***Note:*** The terms “upregulated” and “downregulated” are not appropriate for untargeted lipidomics alone, as they imply transcriptional or enzymatic regulation. This workflow measures relative differences in lipid abundance between groups. Report features as increased or decreased (or higher or lower) abundance.

### Biological interpretation and reporting

**Timing: Variable (hours to days, depending on dataset size, experience level, and interpretation detail)**

Here, we describe the steps for interpreting and reporting the obtained data.

**CRITICAL:** Interpret untargeted lipidomics datasets within the biochemical constraints of the organism, the sample type, and the workflow limitations. Consider confidence tiers during data interpretation; define the inclusion of low-confidence annotations based on the study design and desired level of stringency. Confirm biologically relevant or unexpected lipid features using authentic standards where possible.

96. Prioritize biological conclusions derived from high-confidence lipid annotations supported by MS/MS evidence or authentic standards.

***Note:*** Use mass-matched features (low-confidence annotation tier) as provisional, low-confidence assignments and treat them as supporting context rather than primary evidence, since accurate mass alone does not resolve isomers and can inflate false positives.

97. Interpret lipid changes in the context of known biochemical pathways and matrix-specific lipid biochemistry.

***Note:*** Emphasize pathway-consistent patterns, and drill down to molecular species when structural evidence and analytical performance support that resolution. Avoid mechanistic claims that exceed the structural certainty of the annotation tier.

**CRITICAL:** Multivariate models and feature-ranking approaches can highlight patterns in the data, but do not establish causality. Biological interpretation should focus on reproducible trends and should prioritize features supported by strong analytical evidence.

98. When reporting results, fully document the experimental design and all analytical decisions.

***Note:*** This includes sample handling and storage times, extraction and resuspension conditions, LC–MS settings for each polarity, injection order, blank and QC placement, calibration and recalibration steps, filtering thresholds, imputation rules, normalization strategy, drift correction criteria, statistical tests, multiple-testing correction, and any data transformations or scaling. State which datasets were used for each analysis, such as with or without QCs.

## Expected outcomes

This protocol describes how to generate high-quality, reproducible untargeted LC-MS lipidomics datasets from 5 μL of human serum. Following completion of data acquisition, processing, and filtering, users can expect to obtain curated feature tables containing lipid species annotated by MS/MS spectral matching and tentatively assigned features supported by mass matching (Table S3). As general acceptance criteria for data quality, pooled QC injections should exhibit internal-standard RSD values below 30%, mass accuracy within ±3 ppm, and retention-time reproducibility within ±5 s.

Application of this workflow to human serum typically yields over 300 high-confidence annotations (MS/MS matches supported by CCS) and >500 mass-matched annotations, spanning major lipid categories, including glycerophospholipids, glycerolipids, sphingolipids, sterol lipids, and fatty acyls; however, the number of annotations may vary depending on biological replicates, biochemical interventions, grouping parameters, and sample compositions. In the example dataset shown in this protocol, 563 MS/MS-supported annotations and 2,288 mass-matched annotations were obtained from commercial human serum (Figure 8). Table S3 represents the final annotated results for the example dataset; confidence tiers are provided to enable user-defined filtering for downstream analysis. The values reported in this protocol, however, reflect the example dataset used to demonstrate the workflow and should not be interpreted as a general expectation. Even when applying the same protocol and acquisition parameters, variation in annotation counts arises from differences in sample composition, lipid abundance, and biological state (e.g., disease, intervention, or physiological condition).

The final outputs of this protocol include normalized data tables containing lipid annotations and relative intensity values (see Table S3 for an example). These datasets are suitable for additional univariate and multivariate statistical analyses, lipid-class-level summaries, and integration with complementary experimental or omics data.

## Limitations

No single LC–MS method captures the complete lipidome. Extraction biases, chromatographic selectivity, ionization mode, adduct formation, and mass-spectrometric detection biases lead to preferential discovery of certain lipid classes and molecular species. Low-abundance lipids may remain undetected due to extraction efficiency, ion suppression, or instrument sensitivity.

This workflow provides relative quantification using internal standard normalization with a limited number of representative deuterated lipids. Signal intensity (peak height) ratios are used for reliable comparison between lipid abundances. Absolute (molar) concentration requires calibration curves constructed from authentic isotopically labeled standards matched to each analyte in the same matrix and measured under identical analytical conditions. Without these calibration curves, differences in ionization efficiency between lipid species, matrix effects, and detector response variability prevent reliable conversion of signal intensity to concentration. The described strategy applies to this workflow; different analytical applications may have distinct quantitative requirements.

Annotations generated by this workflow generally correspond to the molecular species level (headgroup, fatty acyl composition, and modifications) or summed species level (total carbon and unsaturation). Positions of double bonds, sn-positions of fatty acyl chains, and stereochemistry cannot be confidently assigned without additional structural methods.

This workflow generates putative lipid annotations as part of an untargeted data-processing pipeline. Putative high-confidence lipid annotations rely on accurate mass, retention time, ion mobility, and MS/MS spectral matching, whereas low-confidence annotations are limited to mass and retention time matching. Isomeric lipid species may remain ambiguously annotated or unresolved under untargeted acquisition conditions. Mass matching is used to assign tentative identities to reproducibly detected features lacking confident MS/MS spectral matches. These assignments should be interpreted carefully, as exact mass alone is insufficient to distinguish structurally related lipid species. The annotation workflow includes retention time evaluation, class-specific Kendrick mass defect filtering, CCS trend filtering, removal of redundant or highly correlated features, and a mammalian-specific biological plausibility filter to remove annotations that are inconsistent with expected lipid classes, polarity, or chemically realistic compositions. This includes exclusion of non-mammalian lipid classes, implausible chain lengths or degrees of unsaturation, chemically unrealistic plasmalogens, and known contaminants. Still, tentative annotations generated by mass matching require targeted follow-up experiments for confirmation. Annotations of potentially interesting species should be confirmed with authentic standards whenever possible.

Finally, this protocol emphasizes analytical and biochemical plausibility filtering to reduce false-positive lipid assignments. While conservative filtering improves robustness, it may exclude low-abundance or uncommon lipid species, particularly in small or heterogeneous datasets. This workflow is optimized for discovery lipidomics and hypothesis generation; follow-up targeted assays are required for structural confirmation and absolute quantification of candidate lipid biomarkers.

## Troubleshooting

### Problem 1

Evidence of *ex vivo* lipid degradation, including elevated lysophospholipids, diacylglycerols, oxidized lipid features, or progressive baseline complexity (Steps 18, 68).

### Potential solution

Ensure that all sample handling steps are performed within the defined time windows. Store serum samples at −80°C. Thaw serum samples at 4°C, minimize exposure to oxygen and light, and avoid repeated freeze–thaw cycles. Confirm that extracts are flushed with nitrogen, capped, and stored immediately after drying to limit exposure to oxygen and light. Inspect pooled QC injections and internal standards across the sequence for systematic increases in lysolipids or oxidized features. Such trends indicate handling- or storage-related artifacts rather than biological variation and warrant re-extraction.

### Problem 2

Persistent background features detected in solvent blanks or extraction blanks (Step 62, 68, 70).

### Potential solution

Inspect solvent blanks and extraction blanks to determine whether background signals originate from solvents, consumables, or carryover. Infuse solvents into the mass spectrometer’s ion source using a clean syringe and syringe pump to assess contamination sources. Confirm that all solvents, mobile phases, and calibration solutions were prepared and stored in clean glass containers with PTFE-lined caps, using high-quality, LC-MS grade reagents. Verify consistent use of high-quality, colorless plastic consumables from a single manufacturer and with the same lot number throughout the project. Rinse the LC-MS system for 12 h with 1:1 water/methanol and 100% 2-propanol.

### Problem 3

Low, unstable, or drifting internal standard signal intensities across samples or pooled QC injections (Steps 31, 38, 44, 59, 67, 68, 76).

### Potential solution

Common causes of internal standard signal variation include inaccurate addition or mixing of the internal standard mixture during sample preparation; variability when pipetting the organic phase during extraction; evaporation or adsorption losses during handling; autosampler instability; trapped air bubbles in vial inserts; injection variability; or contamination or partial clogging affecting ionization or chromatographic performance. Verify that the SPLASH internal standard mixture was added accurately to all samples and pooled QC aliquots prior to protein precipitation or extraction. Vortex the internal standard mixture thoroughly before use to ensure homogeneity. Pre-wet pipette tips before dispensing internal standards and organic solvents to minimize volume loss, and use a new tip for each addition. Confirm that samples were mixed adequately after standard addition. Check each vial insert for trapped air and remove any bubbles before placing the vials in the autosampler.

Inspect extracted ion chromatograms of internal standards to determine whether signal changes are systematic across the sequence or restricted to individual injections. Re-inject selected samples or QC injections to assess injection reproducibility. Check vial inserts for sufficient sample amount. Replace vial caps if septa appear damaged.

Verify autosampler performance, including temperature control (4°C–8°C), injection-volume consistency, needle integrity, and needle-wash conditions. Inspect LC backpressure and chromatographic peak shapes for signs of partial clogging or ion suppression that may affect internal standard signals. Examine the electrospray source sprayer using a microscope or a magnifying lens to assess its positioning and detect damage or contamination. If particulate buildup is suspected, sonicate the sprayer tip in 1:1 water/2-propanol and flush at high flow rate to remove deposits before reinstalling. Recalibrate the instrument and evaluate source stability using a tuning mixture.

### Problem 4

Retention-time drift, reduced chromatographic reproducibility, or distorted peaks across injections (Step 59).

### Potential solution

Possible causes include incorrect mobile-phase preparation; inadequate column equilibration; temperature instability; trapped air bubbles in solvent lines or pump head; gradient mixing errors; column aging or partial blockage; or LC system leaks.

Review system backpressure values from previous injections to identify gradual increases or sudden changes indicative of column leaks, aging, or blockages. Confirm accurate preparation of mobile phases, paying close attention to solvent ratios and ammonium formate concentration. Ensure solvents are properly mixed and check for precipitates or turbidity. If retention-time drift persists or exceeds ± 10 seconds, prepare fresh mobile phases, prime and flush the LC system, and repeat column equilibration prior to reinjecting samples. Inspect chromatographic peak shapes and retention stability using internal standards or reference lipids to confirm restoration of chromatographic performance.

If instability persists, verify the column compartment temperature and inspect the LC system for leaks, blockages, or trapped bubbles in the solvent lines. Prime or purge the LC system to remove bubbles. Rinse for 12 h with 1:1 water/methanol and 100% 2-propanol to dislodge precipitates. Replace the column if permanent damage is suspected.

If retention-time drift persists, prepare fresh mobile phases and repeat column equilibration before reinjection. Use a new chromatographic column if needed.

### Problem 5

Unstable, implausible, or overconfident lipid annotations, including inconsistent CCS values, poor MS/MS support, or biologically unlikely lipid classes (Step 78, 84).

### Potential solution

Potential causes include inaccurate mass or ion-mobility calibration; chimeric MS/MS spectra caused by co-eluting, isobaric ions; insufficient collision energies; interference from contamination-derived features; or over-reliance on accurate-mass matching without orthogonal validation.

Confirm successful mass and ion-mobility recalibration before performing lipid annotation by reviewing calibration metrics for all injections. Inspect mass errors, CCS deviations, and retention-time consistency across injections. Check m/z, retention time, CCS values, and adduct formation for internal standards.

Prioritize lipid annotations supported by MS/MS spectra and CCS, and apply accurate-mass matching only after analytical and reproducibility-based filters are satisfied. Evaluate retention-time and ion-mobility coherence within lipid classes and remove features that deviate substantially from expected class-specific behavior. Inspect MS/MS spectra for evidence of chimeric fragmentation, low signal-to-noise, or co-eluting compounds that may compromise spectral interpretation.

Evaluate potential sources of biological contamination, including solvents, plasticware, bacterial growth, and handling practices. Apply biochemical plausibility filters appropriate to the biological matrix to restrict lipid classes and structural patterns unlikely to occur in the studied system. This prevents propagation of unsupported or spurious lipid annotations into downstream statistical analysis and biological interpretation.

## Resource availability

### Lead contact

Further information and requests for resources and reagents should be directed to and will be fulfilled by the lead contact, Adriana Zardini Buzatto (adriana.zardinibuzat@ucalgary.ca).

### Technical contact

Technical questions on executing this protocol should be directed to and will be answered by the technical contact, Adriana Zardini Buzatto (adriana.zardinibuzat@ucalgary.ca).

### Materials availability

This study did not generate new unique reagents.

### Data and code availability

• Original data have been deposited to MetaboLights as MTBLS14002 and are publicly available as of the date of publication. The accession number for the data reported in this paper is Metabolights: MTBLS14002.
• All original code has been deposited at GitHub and is publicly available as of the date of publication (https://github.com/Buzattoresearch/BRG-LipidQuest and https://doi.org/10.5281/zenodo.19545764).
• Any additional information required to reanalyze the data reported in this paper is available from the lead contact upon request.

## Acknowledgments

This work was supported by grants from the 10.13039/501100000038Natural Sciences and Engineering Research Council of Canada (10.13039/501100000038NSERC) and the 10.13039/501100001804Canada Research Chairs (CRC) program. F.S.M.’s salary was partially supported by the University of Calgary’s VPR Postdoctoral Excellence Award. This workflow was developed within the Calgary Metabolomics Research Facility (CMRF) at the University of Calgary, Canada. We thank Bruker Scientific for their support.

## Author contributions

F.S.M.: conceptualization, data curation, methodology, software, visualization, writing – original draft, and writing – review and editing; A.Z.B.: conceptualization, funding acquisition, project administration, resources, software, supervision, and writing – review and editing

## Declaration of interests

The authors declare no competing interests.

## Declaration of generative AI and AI-assisted technologies in the writing process

During the preparation of this work, the authors used ChatGPT in order to assist with Python code preparation and troubleshooting in BRG LipidQuest. After using this tool/service, the authors reviewed and edited the content as needed and take full responsibility for the content of the published article.

## Supplemental information

Table S1. Example of an exported feature table from MetaboScape used as input for downstream data processing with the Buzatto Research Group LipidQuest routine ((https://github.com/Buzattoresearch/BRG-LipidQuest, DOI: 10.5281/zenodo.19545764) (positive ionization mode), related to Step 79 This table illustrates the structure and content of the software output prior to downstream filtering and normalization and does not represent a final dataset, which is exemplified in Supp. Table S3.

Table S2. Example of an exported feature table from MetaboScape used as input for downstream data processing with the Buzatto Research Group LipidQuest routine (https://github.com/Buzattoresearch/BRG-LipidQuest, DOI: 10.5281/zenodo.19545764) (negative ionization mode), related to Step 79 This table illustrates the structure and content of the software output prior to downstream filtering and normalization and does not represent a final dataset, which is exemplified in Supp. Table S3.

Table S3. Example of the final annotated feature spreadsheet generated by the Buzatto Research Group LipidQuest routine (https://github.com/Buzattoresearch/BRG-LipidQuest, DOI: 10.5281/zenodo.19545764), related to Step 91 Lipid annotations are reported with associated annotation confidence tiers (column Q) reflecting the level of supporting evidence. High-confidence annotations are supported by MS/MS spectral matching, accurate mass, retention time, and ion mobility (CCS), whereas lower-confidence annotations are based on accurate mass and retention time. All features have been subjected to quality control and filtering steps, including retention time evaluation, adduct consistency, and biological plausibility screening. Confidence tiers are provided to guide interpretation and enable user-defined filtering for downstream analysis. This table represents the final dataset and can be used for statistical analysis; confidence tiers are provided to enable user-defined filtering depending on the desired level of stringency. The annotations and values reported here reflect the example dataset used in this protocol and should not be interpreted as a fixed expectation for serum lipidomics.
